# A Systematic Review on Retinal Biomarkers to Diagnose Dementia from OCT/OCTA Images

**DOI:** 10.3233/ADR-230042

**Published:** 2023-11-01

**Authors:** Yehia Ibrahim, Jianyang Xie, Antonella Macerollo, Rodolfo Sardone, Yaochun Shen, Vito Romano, Yalin Zheng

**Affiliations:** a Department of Eye and Vision Sciences, University of Liverpool, Liverpool, UK; b Department of Pharmacology and Therapeutics, Institute of Systems, Molecular and Integrative Biology, University of Liverpool, Liverpool, UK; c Department of Neurology, The Walton Centre NHS Foundation Trust, Liverpool, UK; d Statistics and Epidemiology Unit, Local Healthcare Authority of Taranto, Taranto, Italy; e Department of Electrical Engineering and Electronics, University of Liverpool, Liverpool, UK; f Department of Medical and Surgical Specialties, Radiological Sciences, and Public Health, University of Brescia, Brescia, Italy; g Liverpool Centre for Cardiovascular Science, University of Liverpool and Liverpool Heart and Chest Hospital, Liverpool, UK

**Keywords:** Alzheimer’s disease, dementia, mild cognitive impairment, neurodegenerative disorders, optical coherence tomography, optical coherence tomography angiography, retinal biomarkers

## Abstract

**Background:**

Traditional methods for diagnosing dementia are costly, time-consuming, and somewhat invasive. Since the retina shares significant anatomical similarities with the brain, retinal abnormalities detected via optical coherence tomography (OCT) and OCT angiography (OCTA) have been studied as a potential non-invasive diagnostic tool for neurodegenerative disorders; however, the most effective retinal changes remain a mystery to be unraveled in this review.

**Objective:**

This study aims to explore the relationship between retinal abnormalities in OCT/OCTA images and cognitive decline as well as evaluating biomarkers’ effectiveness in detecting neurodegenerative diseases.

**Methods:**

A systematic search was conducted on PubMed, Web of Science, and Scopus until December 2022, resulted in 64 papers using agreed search keywords, and inclusion/exclusion criteria.

**Results:**

The superior peripapillary retinal nerve fiber layer (pRNFL) is a trustworthy biomarker to identify most Alzheimer’s disease (AD) cases; however, it is inefficient when dealing with mild AD and mild cognitive impairment (MCI). The global pRNFL (pRNFL-G) is another reliable biomarker to discriminate frontotemporal dementia from mild AD and healthy controls (HCs), moderate AD and MCI from HCs, as well as identifing pathological Aβ_42_/tau in cognitively healthy individuals. Conversely, pRNFL-G fails to realize mild AD and the progression of AD. The average pRNFL thickness variation is considered a viable biomarker to monitor the progression of AD. Finally, the superior and average pRNFL thicknesses are considered consistent for advanced AD but not for early/mild AD.

**Conclusions:**

Retinal changes may indicate dementia, but further research is needed to confirm the most effective biomarkers for early and mild AD.

## INTRODUCTION

Currently, more than 55 million people worldwide [1] and 850,000 people in the UK [2] are living with dementia. Globally, there are 10 million newly recorded dementia cases annually [1]. Patients with dementia suffer deterioration of cognitive functions that affect their independence to perform basic daily activities without the assistance of caregivers. The approximated global cost of dementia is $1.3 trillion in 2019, and noteworthy that these expenses are projected to reach $2.8 trillion by 2030 [1]. In the UK, the total health care cost is expected to increase from currently £34.7billion to £94.1billion by 2040 [2]. The current methods of detecting dementia in clinic include cerebrospinal fluid (CSF) analysis, brain imaging, blood tests, and genetic testing [3]. A brain magnetic resonance imaging (MRI) scan could take from 15 min and up to 90 min [4], while CSF analysis requires an invasive procedure to be performed [5]. Though these methods are effective in evaluating cognitive functions, they are costly, difficult to obtain samples for CSF analysis, and time consuming for brain imaging. Hence these methods are not ideal as screening tool for mild cognitive impairment (MCI), and/or early Alzheimer’s disease (AD). On the other hand, the retina is part of the central nervous system (CNS), and hence retinal changes may indicate neurodegenerative processes [6, 7].

One major aim of this review is to investigate whether optical coherence tomography (OCT) and OCT angiography (OCTA) imaging could provide researchers with biomarkers for the purpose of identifying cognitive impairments. Therefore, the objectives of this research are: 1) discuss OCT/OCTA retinal imaging techniques and their extracted parameters that could potentially be utilized as a screening tool to assess cognitive functions, 2) explore briefly some basic methods to extract OCT/OCTA parameters, 3) study various retinal alterations and their association with brain degeneration, 4) investigate which retinal biomarkers hold the potential to diagnose neurodegeneration disorders, 5) challenge the reliability of biomarkers extracted around the macula and optic disc in discriminating between patients with neurogenerative disorders and healthy individuals, and 6) discuss briefly the recent development in using retinal biomarkers to build classification models for the purpose of identifying neurodegenerative disorders, hence, emphasizing the diagnostic value for retinal biomarkers.

## METHODS

**Fig. 1:**
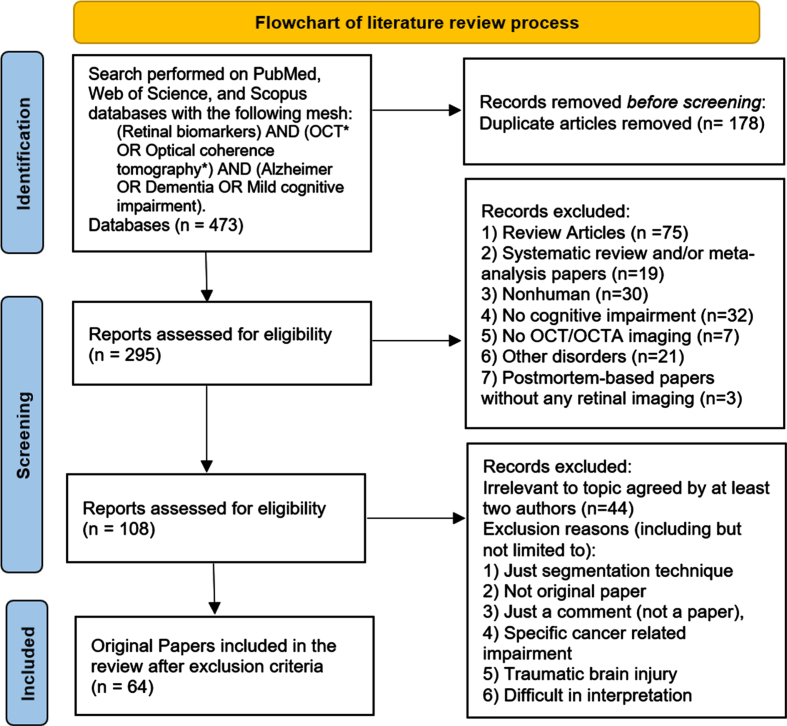
Literature review process flowchart.

A search was performed on PubMed, Web of Science, and Scopus following Preferred Reporting Items for Systematic Reviews and Meta-Analyses (PRISMA) guidelines [8], and the following keywords were used: (Retinal biomarkers) AND (OCT* OR Optical Coherence Tomography*) AND (Alzheimer OR Dementia OR Mild Cognitive Impairment). The search started from June 6, 2022 through December 21, 2022 resulted in 218, 170, and 85 research articles from PubMed, Web of Science, and Scopus, respectively. A total of 178 articles were removed from the total 473 articles for repetition, and hence, 295 were left for screening. The 295 research articles were composed of 75 review articles, 19 systematic review and/or meta-analysis papers, and 201 papers categorized as original papers. Since the present research is exclusively centered on original papers, the 94 review/systematic review and/or meta-analysis papers were excluded. Another 133 papers were also excluded, which involved 30 nonhuman articles, 32 papers with no cognitive impairment, 7 papers not including either OCT or OCTA imaging, 3 postmortem-based papers without any retinal imaging, and 21 paper with other disorders (including but not limited to: 4 Huntington’s disease, 9 multiple sclerosis, 7 Parkinson’s disease (PD) without cognitive impairment, 1 cerebral small vessel disease). Two reviewers (YI and JX) reviewed and selected papers independently and then consensus was made after discussions. Briefly, a total of 228 articles were left out from this systematic review, hence, 64 original papers were included. The full process is illustrated in Fig. 1.

This review also followed the steps provided by [9] to a perform quality assessment, where papers are classified into low, good, and high qualities denoted by LQ, GQ, and HQ, respectively. The assessment grades of all included papers in this review could be found in the results section.

## RESULTS

The results section will start by explaining different OCT and OCTA imaging techniques and the layers definitions currently adopted in the literature. In addition, OCT/OCTA parameters as well as the currently available methods used to extract such parameters will be unraveled. Next, a summary of the included studies in this review will be listed in a table. This summary table includes sample size, the cognitive decline diagnosis, the participants’ age and their eye health status, the used imaging device(s), the explored retinal parameters, and finally the studies are categorized to either cross-sectional or longitudinal.

### OCT/OCTA imaging techniques and retinal layers definitions

**Fig. 2:**
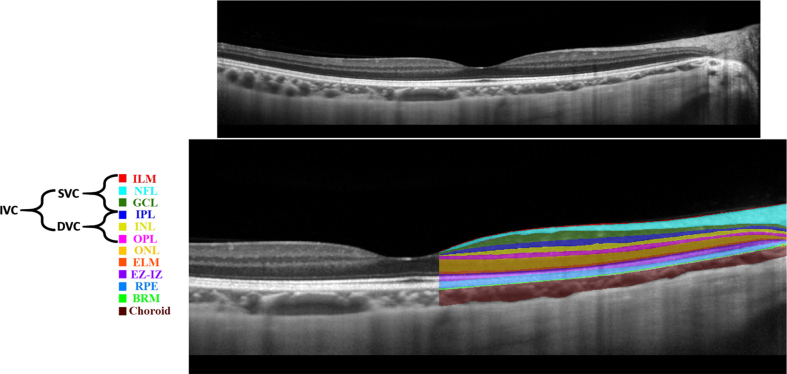
Retinal layers in a spectral domain optical coherence tomography b-scan image: unlabeled (top), labelled (bottom).

Various imaging techniques could be leveraged to capture retinal changes. Nowadays, non-invasive imaging techniques such as OCT and OCTA are used to capture structural and vascular changes in the retina, respectively [10]. OCT is based on interference principles and can be categorized into Time domain (TD)-OCT and Frequency domain (FD)-OCT. The FD-OCT contains Spectral-Domain OCT (SD-OCT) and Swept-Source OCT (SS-OCT) [11]. FD-OCT has higher acquisition speed and better resolution replacing TD-OCT [11]. SD-OCT is commonly used in ophthalmology clinics; however, SS-OCT is a new technology with higher imaging speed and better visualization of deeper structures due to the use of longer wavelength light source [11]. SS-OCT uses more invisible light source than SD-OCT, hence, SS-OCT is relatively more comfortable for patients [11]. An example of an SD-OCT (B-scan) is shown in Fig. 2 (top). OCT can almost resolve all the retinal cellular layers as demonstrated in Fig. 2 (bottom). From the inner to outer, layers include inner limiting membrane (ILM), nerve fiber layer (NFL), ganglion cell layer (GCL), inner plexiform layer (IPL), inner nuclear layer (INL), outer plexiform layer (OPL), outer nuclear layer (ONL), external limiting membrane (ELM), ellipsoid zone and interdigitation zone (EZ-IZ), retinal pigment epithelium (RPE), and Bruch’s membrane (BRM).

The early OCTA segmentation by [12] defined the superficial (SCP) and the deep capillary plexuses (DCP), respectively, based on the retinal anatomical layers. Simply, the SCP was defined from ILM to IPL, whereas the DCP was defined from the outer boundary of the IPL to OPL [12]. Of note, this SCP and DCP segmentation approach is commonly used by the Heidelberg SD-OCT machine [13].

**Fig. 3:**
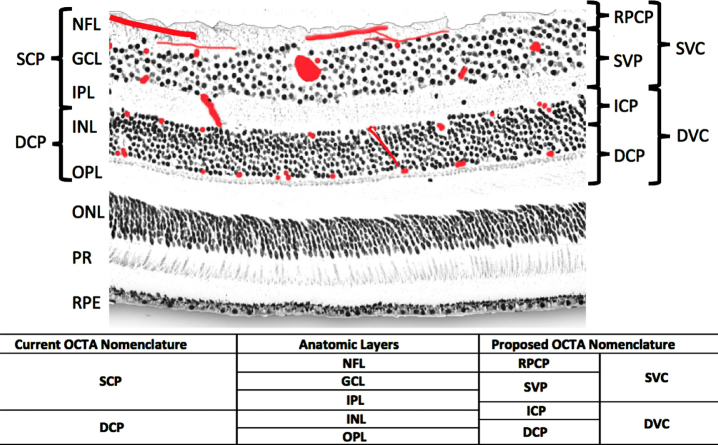
Anatomic layers, previous and new optical coherence tomography angiography (OCTA) segmentation names [15]. PR, photoreceptor layer.

Previously, in [14], the macula was analyzed into four retinal vascular networks namely: radial peripapillary capillary plexus (RPCP), superficial vascular plexus (SVP), intermediate capillary plexus (ICP), and DCP as shown in Fig. 3. The SVP was segmented between the inner 80% of the ganglion cell complex (GCC), where GCC consists of NFL+GCL+IPL. The ICP was defined as the outer 20% of the GCC to the inner 50% of the INL, whereas the DCP was defined between the outer 50% of the INL and the OPL. Recently [15], new scientific names based on segmentation boundaries were proposed where superficial vascular complex (SVC) is composed of RPCP and SVP, while deep vascular complex (DVC) is composed of ICP and DCP as shown in Fig. 3. Nevertheless, the combination of both SVC and DVC (SVC+DVC) is denoted by the inner vascular complex (IVC), as shown in Fig. 2 (bottom).

In order to provide a clear explanation of the various retinal layer segmentations and layer definitions in the literature, Table 1. demonstrates diverse OCT machines along with the appropriate layer definitions.

**Table 1:** Layer definitions by different OCT machines

	SCP (ILM to IPL), DCP (INL to OPL)	SCP (ILM to GCL), ICP (IPL to INL), DCP (INL to OPL)	SVP (ILM to 10μm above IPL), DVP (10μm above IPL and 10μm below OPL	SCP (3μm below ILM and 15μm below IPL), DCP (between 15 and 70μm below IPL till OPL)
A-SD [16–18]	√	×	×	×
A-SD [19, 20]	×	×	√	×
A-SD [21]	×	×	×	√
H-SD [22]	×	√	×	×
H-SD [23]	√	×	×	×
C-HD [24–26]	√	×	×	×

H-SD, Heidelberg SD-OCT; C-HD, Cirrus HD-OCT; A-HD, RTVue-XR Avanti SD-OCT

**Fig. 4:**
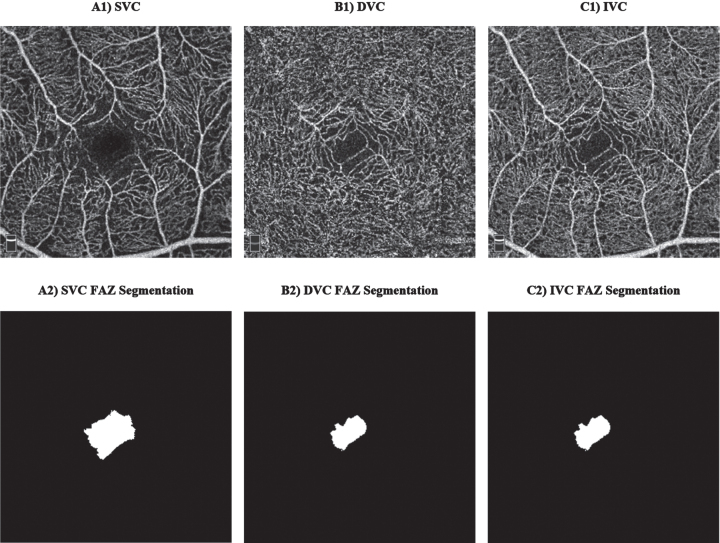
Examples of optical coherence tomography angiography (OCTA) scans: A1) superficial vascular complex (SVC), B1) deep vascular complex (DVC), C1) inner vascular complex (IVC), and their corresponding manual foveal avascular zone (FAZ) segmentation.

OCTA is a new emerging imaging technique that can be used to resolve retinal vasculature at the capillary level in a non-invasive manner. The En Face OCTA examples in Fig. 4 are image projections in 2D originated from a volumetric 3D OCTA around the fovea. Notably, there are various options when performing image projections at different retinal layers depths around the fovea, for instance, SVC, DVC, and IVC projections examples could be found in Fig. 4 (A1-C1). Briefly, to extract vascular parameters around the fovea, a series of steps are taken to obtain an OCTA En Face image. Firstly, a selection of retinal layers is made, for instance, from the ILM to the OPL to obtain the IVC. Next, the volumetric 3D OCTA of the selected layers is projected into a 2D image known as the En Face OCTA. This projected image will include the vessels and microcapillaries to be analyzed, providing a clear and detailed view which is ready for the extraction of vascular parameters.

### OCT/OCTA parameters

Interesting vascular changes in the retina could be observed at SVC and DVC; however, we shall report the old scientific retinal segmentation boundaries names since most of the previously published works reported the results of SCP, DCP, SVP, and ICP. These changes could be evaluated and categorized into vascular and foveal avascular zone (FAZ) changes, where manual FAZ extraction examples are shown in Fig. 4 (A2-C2). Important vascular changes could be captured via vascular density (VD) or vascular perfusion density (VPD), fractal dimension (FD), and vessel length density (VLD). Yoon and colleagues [27] defined VD/VPD as the ratio between the number of pixels in the perfused retinal vascular area to the number of pixels of the entire retina. VLD is a ratio that is defined between the total length occupied with blood vessels in an area and the total area [16]. FD is a non-linear analysis which aims to measure the geometric complexity of vessels via box counting analysis [28–30].

**Fig. 5:**
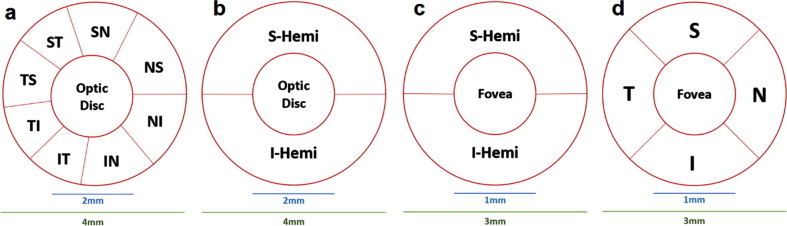
HD Angio Disc mode (optic disc) Report Layout Legend: a) Garway-Heath (GH) map [31]; b) Hemisphere Maps; 2) Angio Retina mode (macular) Report Layout Legend: c, d) Hemisphere and Quadrant Maps [32].

Generally, the process of scanning around the optic nerve head is called a disc scan mode, which could be partitioned into upper and lower sectors namely superior-hemi (S-Hemi) and inferior-hemi (I-Hemi) sectors, respectively, as shown in Fig. 5b. Furthermore, Garway-Heath et al. [31] proposed another sectioning variant in Fig. 5a, and these sections are named: superotemporal, superonasal, inferotemporal, inferonasal, nasal superior, nasal inferior, temporal superior, and temporal inferior, and denoted as ST, SN, IT, IN, NS, NI, TS, and TI, respectively. Similarly, variations around the fovea is considered as a region of interest [16, 22]; however, some papers focused on some specific regions around the fovea [21, 32–34]. As shown in Fig. 5d, a scan around the fovea could be divided into sections namely: superior (S), inferior (I), nasal (N), and temporal (T). Another simpler splitting variation around the fovea is shown in Fig. 5c with S-Hemi and I-Hemi sections.

**Fig. 6:**
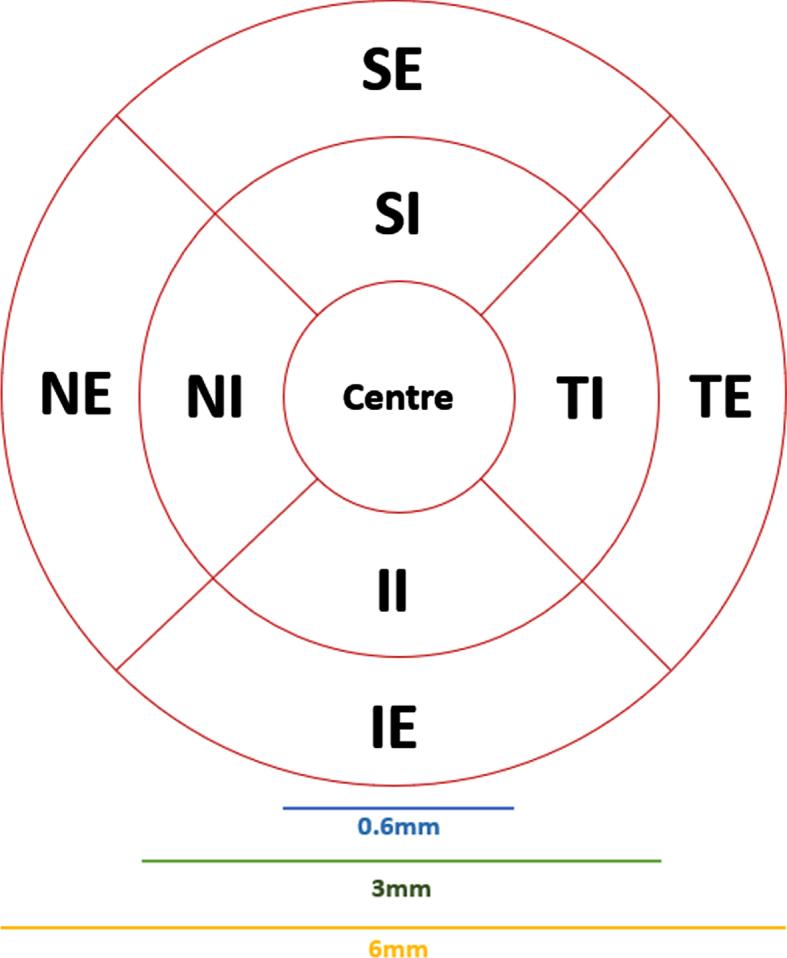
Microvascular network sections in superficial capillary plexuses (SCP) (adapted from [21]).

As illustrated in Fig. 6, the four quadrants partitions around the fovea follows a comparable pattern showed/explained previously in Fig. 5d. Additionally, SI, II, TI, and NI belongs to the inner ring; whereas SE, IE, TE, and NE belongs to the external ring around the center fovea.

### Extracting OCT/OCTA parameters methods

Some research works use the commercial software which accompanies the OCT/OCTA machine. For instance, both [19, 35] extracted OCT and OCTA parameters automatically by a commercial RTVue XR software and Optovue AngioVue system (software ReVue XR). In addition, the AngioAnalyticstrademark software was used by [36] to compute VD and FD for retinal vasculature as well as the perimeter and Acircularity Index for FAZ. Noteworthy, the Acircularity Index was introduced in [37] such that it measures the level of FAZ shape abnormalities. In the study by Chua et al. [38], Cirrus Review software generated both macular and optic disc images, whereas Iowa Reference Algorithm (IRA) [39–41] was used to automatically segment individual retinal layers. The optic disc and macular images were stitched and followed by a segmented retinal vessel map obtained by a customized built algorithm, by [38], using MATLAB software. Furthermore, Jáñez-Escalada and colleauges [42] also used IRA [39–41] as a retinal layer segmentation module (Iowa Reference Algorithms 3.6 Retinal Image Analysis Lab, Iowa Institute for Biomedical Imaging, Iowa City, IA, USA). On the contrary, some other works attempted to use deep-learning methods to develop retinal vessels as well as retinal layers segmentation methods. Based on U-Net introduced in [43], the research by [44] created a publicly available ROSE dataset and built a custom tool built specifically for the retinal vessel segmentation task of OCTA angio scans. Another work by [45] aimed to segment intraretinal layers using a cascaded version of U-Nets. In [45], the segmented layers were: ILM, mRNFL, GCL, IPL, GCIPL/GCC, INL, OPL, ONL, ELM, myoid zone, ellipsoid zone (EZ), outer segment (OS), RPE, outer layer, and Bruch’s membrane. A different study by [46] attempted to generate super-resolved segmented retinal layers using Generative Adversarial Network-based approach.

### Overview of the included studies

A summary of all the longitudinal and cross-sectional (CS) studies included in this review as well as the cohort’ details, study type, imaging methods, and studied biomarkers are found in Table 2. The results are categorized into mainly structural and vascular parameters which are split cross-sectionally and longitudinally. The CS results of structural parameters include subcategories involving optic disc and macula related parameters. On the contrary, the structural and vascular parameter of the longitudinal studies are combined sequentially due to the limited number of works. Additionally, Table 3 includes the details of CS pRNFL related parameters, while Table 4 includes the longitudinal pRNFL related parameters. However, the tables for CS macula related parameters (Supplementary Table 1), CS vascular parameters (macula and optic disc) (Supplementary Table 2), and longitudinal macula-related parameters (Supplementary Table 3) are found in the Supplementary Material due to the complexity of the analyzed data. Noteworthy, all tables illustrate significant changes by ↑ or ↓ arrows, insignificant changes by -^*NS*^, and –for any missing parameters. Moreover, another subsection will discuss research works which attempted to build classification models based on statistically significant biomarkers.

**Table 2:** Summary of the studies included in this review

Paper	Sample Size (N) &Diagnosis	Mean age±std in years	S tudy Type	Studied layers and/or Retinal biomarkers	Eye Diseases (Included/ Excluded)	Imaging Methods	QA
[47]	Mild AD: 19, HCs: 24	Mild AD: 79.16±3.93, HCs: 75.71±2.83	CS	The thicknesses of mRNFL, GCL, IPL, INL, OPL, ONL, IS/OS, OSL, OPR, RPE, Total Retina	Excluded	T-SD, D-Fundus, Brain MRI	LQ
[42]	Mild AD: 19, HCs: 24	Mild AD: 79.16±3.93, HCs: 75.71±2.83	CS	The thicknesses of mRNFL, GCL, IPL, INL, OPL, ONL, IS/OS, OSL, OPR, RPE, Total Retina.	Excluded	T-SD	LQ
[16]	aMCI: 13, eAD: 3, HCs: 16	aMCI/eAD: 73.03±8.24, HCs: 73.60±7.69	CS	Parafoveal SCP, Peripapillary RPC, Peripapillary SVC; Vascular parameters: VD, Microcapillary VD, VLD, AFI; Structural parameters: pRNFL (G and S) thicknesses, IVC-FAZ	Excluded	A-SD	GQ
[48]	Sample I: 3289 individuals Sample II: 2998 individuals	Sample 1 : 68.9±9.9, Sample 2 : 68.2±9.9	L	pRNFL and macular GC-IPL	Excluded	T-SD	LQ
[21]	MCI: 21, AD: 18, HCs: 21	MCI: 67.81±5.96, AD: 69.94±6.39, HCs: 68.67±5.85	CS	FAZ IVC, VD of SCP and DCP in 4 inner sections: TI, SI, NI, II, and 4 outer sections: TE, SE, NE, IE	Excluded	A-SD	GQ
[22]	MCI due to AD: 24, HCs: 13	MCI-AD: 72.1±6.4, HCs: 73.6±3.7	CS	SCP-VD, ICP-VD, DCP-VD, CC-VD, SCP-VLD, ICP-VLD, DCP-VLD, CC-VLD, FD, SCP-FAZ	Excluded	H-SD	GQ
[34]	AD: 19, HCs: 20	AD: 73.79±7.22, HCs: 74.35±6.07	CS	FRT (center, S, I, T, N quadrants), and mRNFL+GCL (average, I, S, T, N quadrants). VB, VC, and VD were also measured.	No Eye Info	C-HD	LQ
[49]	AD: 9, MCI: 9, HCs: 14	AD: 73.3, MCI: 76.3, HCs: 63.3, std: NA	CS	pRFNL thickness and differences in quadrant thickness	Excluded	C-HD	GQ
[33]	PPG: 54, aMCI: 54, HCs: 54	PPG: 72.29±7.05, aMCI: 73±6.6, HCs: 72.66±7.05	CS	GCC, pRNFL, and VD of RPC plexus in various retinal regions	Included PPG	A-SD	GQ
[50]	MCI: 324, Dementia: 38^*M^, HCs: 613	MCI: 74.2±5.6, Dementia: 77.2±5.6, HCs: 72.4±5.3	CS	The thicknesses of mRNFL, GC-IPL, GCC (mRNFL+GC-IPL), FRT at quadrants (S, I, T, N) in both inner and outer rings, and center of FRT. Global pRNFL thickness, pRNFL at quadrants (S, I, T, N)	Excluded	N-Fundus, N-SD, N-Refract	LQ
[32]	Mild AD: 37, HCs: 29	Mild AD: 63.89±9.574, HCs: 60.28±7.096	CS	Optic disc parameters: pRNFL (overall, GH in S &I Hemi and GH in 8 sections); VD of RPC (whole and peripapillary region, GH in S &I Hemi and GH in 8 sections). Macular parameters: whole foveal thickness, macular thickness (S, I, T, N) and GH in S &I Hemi; VD of SCP and DCP each at whole fovea, total parafovea, parafovea S &I hemi, and parafovea in four quadrants.	Excluded	A-SD	GQ
[51]	Mild AD: 36, HCs: 36	Mild AD: 72.0±7.3, HCs: 71.7±6.0	L	pRNFL thickness (total, S, I, T, N) changes over the 12 months	Excluded	H-SD, D-Fundus, Brain MRI	GQ
[52]	Initially: AD: 42, aMCI: 26, HCs: 66, follow-up 2 years: *MCI*_*AD*_: 9, *MCI*_*MCI*_: 12	AD: 76.8±8.7, aMCI: 74.7±7.8, HCs: 73.8±7.5	L	pRNFL (Avg, S, I, T, N), GC-IPL (mean, min, ST, S, SN, IN, I, IT), central MRT, macular cube volume, mean macular cube thickness	Excluded	C-HD	GQ
[38]	AD: 62, MCI: 108, HCs: 55	AD: 73.3±8.7, MCI: 73.4±6.3, HCs: 71.0±4.7	CS	cpRNFL thickness; Compensated cpRNFL thickness; 10 macular layers: mRNFL, GCL, IPL, INL, OPL, ONL, IS/OS, OSL, OPR, RPE	Excluded	C-SD, C-Refract	GQ
[53]	Group 1^*a^: AD: 43, MCI: 37, HCs: 57; Group 2^*b^: AD: 21, MCI: 18, HCs: 18	AD: 70.94±7.23, MCI: 72.83±6.03, HCs: 69.47±6.90	CS	pRNFL thickness (G, S, I, T, N), macular volume of GCL, IPL, and INL	Excluded	H-SD	HQ
[54]	eAD: 40, HCs: 40	AD: 69.3±4.9, HCs: 68.9±5.1	CS	Mean pRNFL, T, N, I, and S quadrants pRNFL thicknesses	Excluded	SD-OCT ℵ	LQ
[55]	mmAD: 21, HCs: 21	mmAD: 73.1±6.9, HCs: 70.3±7.3	CS	SFCT, ChorT at 500 &1500 microns in the S, I, T and N quadrants. Retinal thickness; peripapillary RNFL average thickness, and in the areas of S, I, T, and N quadrants	Excluded	H-SD	GQ
[56]	PCA: 25, tAD: 23, HCs: 70	PCA: 67.0±7.1, tAD: 64.5±6.8, HCs: 66.3±7.7	CS	pRNFL thickness and total macular thickness (mRT).	Excluded	O-SD	GQ
[57]	AD: 20, PD: 28, HCs: 27	AD: 66.3±6.8, PD: 63.4±6.6, HCs: 64.1±7.1	CS	Macular thickness of mRNFL, GCL, IPL, INL, OPL, and ONL. The FRT from the ILM to the top of the RPE	Excluded	C-SD	GQ
[58]	AD: 57^*A+^, HCs: 85^*A-^	AD: 65.0±7.6, HCs: 67.93±9.4	CS	mRNFL, GCL, and IPL thicknesses in the inner and outer rings. the thicknesses of mean/global pRNFL and pRNFL in T, TS, NS, N, NI, and TI sectors	Excluded	H-SD	GQ
[59]	Initially: MCI:20, HCs: 58 Follow-up: HCs-to-MCI: 8, *HCs* _ *Stable*: 50, *MCI*_*Stable*_ : 10, *MildAD*_*FromMCI*_: 9 *ModerateAD*_*FromMCI*_: 1 *Stable* _ *Total*:60, *Converted* _ *Total*: 18	Stable: 74.13±3.75, Converted 75.33±4.06	L	Average pRNFL thickness (*μm*). pRNFL thickness (*μm*) in S, I, N, and T quadrants	Excluded	C-HD	GQ
[60]	*BIOM*_-*ve*_: 11, *BIOM*_+*ve*_: 9	*BIOM*_-*ve*_: 75.21±4.13, *BIOM*_+*ve*_: 76.29±4.66	L	IVC FAZ area (*mm*^2^) and the annual change of FAZ area	Excluded	A-SD	GQ
[35]	Initially: aMCI: 19, HCs: 18 Follow-up: aMCI-to-Dem: 7, *aMCI*_*Stable*_: 12	aMCI: 75±5.6 years, HCs: 75±6.2 years	L	pRNFL, GCC thicknesses. FAZ area, VD of SCP, DCP, CC, RPC	Excluded	A-SD	GQ
[61]	mmAD: 56, HCs 56	mmAD: 74.0±8.1, HCs: 76.4±8.4	CS	Mean pRNFL thickness, quadrant RNFL thickness (S, I, T, N), thickness at the 12 clock hours of 30° pRNFL were analyzed	Excluded	C-HD	GQ
[62]	MCI: 24, AD: 23, HCs: 43	AD/MCI: 73.4 ± 8.9, HCs: 70.7 ± 6.9	CS	CRT, pRNFL G and TS, NS, N, NI, TI, T thicknesses	Excluded	H-SD	GQ
[63]	amnestic MCI: 59, non-amnestic MCI: 17, HCs: 56	non-amnestic MCI: 70.48±8.01, amnestic MCI: 72.16±7.01, HCs: 68.07±8.93	CS	RNFL, GC-IPL, CST, SFCT thicknesses FAZ Area SCP, Avg and Inn R of both VD and VLD in SCP	Excluded	C-HD	GQ
[25]	AD: 7, HCs: 8	AD: 82.4±6.8, HC: 76.3±11.9	CS	GC-IPL thickness, VD of SCP, DCP, CC	Excluded	C-HD	GQ
[64]	AD: 17, HCs: 22	AD: 71.9±6.6, HC: 68.6±8.4	L	Average pRNFL thickness (*μm*). pRNFL thickness (*μm*) in the following quadrants: S, I, N, and T	Excluded	A-SD	GQ
[65]	aMCI: 23, HCs: 24	aMCI: 67.43 ± 7.07, HCs: 64.58 ± 9.48	CS	Average pRNFL thickness, and pRNFL thickness in quadrants S, I, N, and T. Average FMT, and FMT in the inner and outer ring in quadrants S, I, N, and T, and FMT in fovea. global macular thickness (GLMT) of RNFL, GC-IPL, GCC. 9 ETDRS sectors thicknesses for mRNFL, GC-IPL, GCC.	Excluded	T-SS	GQ
[18]	CI: 268, HCs: 1287	CI: 58.3±8.3 HCs: 51.0±7.8	CS	VD of SCP Fovea, SCP Parafovea, SCP S, SCP I, SCP T, SCP N, DCP Fovea, DCP Parafovea, DCP S, DCP I, DCP T, DCP N. DCP FAZ *mm*^2^, DCP PERIM, mm, DCP Aci-Index, FD300 VAD, FD300 VLD. Thickness of GCC Fovea, GCC Parafovea, GCC S, GCC I, GCC T, GCC N	Excluded	A-SD	GQ
[66]	MCI: 15, mmAD: 15, HCs: 18	MCI: 73.07±9.06, mmAD: 74.20±8.98, HCs: 75.17±5.92	CS	Macula GC-IPL G, Macula NFL G, ONH GC-IPL G, ONH NFL G, ONH NFL NS, ONH NFL N, ONH NFL NI, ONH NFL TS, ONH NFL T, ONH NFL TI	Excluded	H-SD	GQ
[67]	aMCI: 192, AD: 324, HCs: 414	aMCI: 76.46±7.14, AD: 78.99±7.87, HCs: 65.93±9.01	CS	Mean macular thickness (μm), Mean macular volume (*μm*^3^), GCL Width, Mean Macular RNFL width, ETDRS Centre, ETDRS Inn-T, ETDRS Inn-S, ETDRS Inn-N, ETDRS Inn-I, ETDRS Out-T, ETDRS Out-S, ETDRS Out-N, ETDRS Out-I	Excluded	T-3D	GQ
[20]	ATD: 26, HCs: 26	ATD: 74.23±7.55, HCs: 72.58±6.28	CS	VD-SVP, IVC-FAZ, ChorT, outer and ChorT flow rate	Excluded	A-SD	GQ
[68]	AD: 12, MCI: 12, HCs: 32	AD: 72.9±7.2, MCI: 76.3±6.9, HCs: 71.6±5.9	CS	OCT params: Avg mRNFL (μm), Avg GCL (μm), Avg IPL (μm), Avg GCC (μm), Avg macular (μm) in Sectors: C 1-mm, S 3-mm, T 3-mm, I 3-mm, N 3-mm, Total 3-mm, S 6-mm, T 6-mm, I 6-mm, N 6-mm, Total 6-mm. OCTA params: VD for 3 mm SVP, 3 mm DVP, 3 mm CC, 3 mm Choroid, 6 mm SVP, 6 mm DVP, 6 mm CC, 6 mm Choroid	Excluded	C-HD &H-SD	GQ
[69]	Initially: AD: 150, HCs: 75 Follow-up (<3 years): AD: 99. Follow-up (≥3 years): AD: 51	AD: 75.33±NA, HCs: 74.79±NA	L	1st: Avg pRNFL (μm), pRNFL NS (μm), pRNFL N (μm), pRNFL NI (μm), pRNFL TI (μm), pRNFL T (μm), pRNFL TS (μm), N/T ratio. 2nd: ILM, pRNFL, GCL, IPL, INL, OPL, ONL, OLM, PhotoR, RPE. (1st and 2nd segmentation methods)	Excluded	H-SD	GQ
[70]	Initially: MCI:20, HCs: 58 Follow-up: HCs-to-MCI: 8, *HCs* _ *Stable*: 50, *MCI*_*Stable*_ : 10, *MildAD*_*FromMCI*_: 9 *ModerateAD*_*FromMCI*_: 1 *Stable* _ *Total*:60, *Converted* _ *Total*: 18	Stable: 74.13±3.75, Converted 75.33±4.06	L	pRNFL G (μm), pRNFL S (μm), pRNFL I (μm), pRNFL T (μm), pRNFL N (μm)	Excluded	C-HD	GQ
[71]	AD: 48^*A+^, HCs: 38^*A-^	AD: 65.4±8.1, HCs: 60.6±5.0	CS	ChorT (μm), VD Inn R, VD Out R, FAZ (*mm*^2^) of SVP	Excluded	H-SD	GQ
[72]	MCI: 66, Dementia: 43 (AD: 17, DLB: 16, VCID: 6, FTD: 4), HCs: 27	MCI: 71.9±8.7, Dementia: 76.9±7.6, HCs: 63.6±10.4	CS	Central Subfield (μm), Macula Volume (*mm*^3^), Average Macula (μm), GCL+IPL (μm), Minimal GCL+IPL (μm), pRNFL (μm)	Excluded	C-HD	GQ
[73]	AD: 35, MCI: 35, HCs: 35	AD: 75.4±6.9, MCI: 74.1±6.3, HCs: 70.2±8.0	CS	$\mathrm{RNFL}\;{\left(\mu m\right)}^{AMB}$	Excluded	C-HD	LQ
[74]	Initially: MCI: 22, HCs: 82 Follow-up: *MCI*_*Stable*_:10 MCI-to-Dem: 10 *HCs* _ *Stable*: 55, HCs-to-MCI: 8 *Stable* _ *Total*: 60, *Converted* _ *Total*: 18	*Stable* _ *Total*: 74.1±3.7 *Converted* _ *Total*: 75.3±4.1	L	pRNFL (μm)	Excluded	C-HD	GQ
[75]	CH-PAT: 27, CH-NAT: 16	CH-PAT: 75.2±8.4, CH-NAT: 74.1±7.9	CS	pRNFL (μm), GC-IPL (μm), Macula (μm)	Excluded	C-SD	GQ
[24]	AD: 24, MCI: 37, HCs: 29	AD: 74.9±6.0, MCI: 77.9±6.4, HCs: 76.7±5.3	CS	SCP-VD, DCP-VD, SCP-FD, DCP-FD	Excluded	C-HD	GQ
[76]	Mild AD: 22, Mod AD: 16, HCs: 62	Mild AD: 77.00±6.15, Mod AD: 73.9±11.5, HCs: 73.9±11.5	CS	RT S, RT Total, RT I, GCL S, GCL Total, GCL I, GCL-SZ1, GCL-SZ2, GCL-SZ3, GCL-SZ4, GCL-SZ5, GCL-IZ1, GCL-IZ2, GCL-IZ3, GCL-IZ4, GCL-IZ5, GCL-*Δ*1, GCL-*Δ*2, GCL-*Δ*3, GCL-*Δ*4, GCL-*Δ*5, RT-SZ1, RT-SZ2, RT-SZ3, RT-SZ4, and RT-SZ5, RT-IZ1, RT-IZ2, RT-IZ3, RT-IZ4 and RT-IZ5, RT-*Δ*1, RT-*Δ*2, RT-*Δ*3, RT-*Δ*4 and RT-*Δ*5	Excluded	H-SD	GQ
[17]	AD: 42, MCI: 48, HCs: 45	AD: 71.40±7.82, MCI: 71.67±8.04 HCs: 68.91±5.88	CS	pRNFL (Avg, S, I), GCC (Avg, S, I), RetMap Inn Avg Peri, RetMap Inn S Peri, RetMap Inn I Peri, RetMap Inn N Peri, RetMap Inn T Peri, RetMap Out Avg Peri, RetMap Out S Peri, RetMap Out I Peri, RetMap Out N Peri, RetMap Out T Peri	Excluded	A-SD	GQ
[77]	AD: 21, HCs: 25	AD: 72±11.1, HCs: 72.3±6.5	CS	pRNFL Avg, pRNFL S, pRNFL I, pRNFL T, pRNFL N, Fovea (μm), Fovea (*mm*^3^), Macula S Inn R (μm), Macula I Inn R (μm), Macula T Inn R (μm), Macula N Inn R (μm), Macula S Inn R (*mm*^3^), Macula I Inn R (*mm*^3^), Macula T Inn R (*mm*^3^), Macula N Inn R (*mm*^3^), Macula S Out R (μm), Macula I Out R (μm), Macula T Out R (μm), Macula N Out R (μm), Macula S Out R (*mm*^3^), Macula I Out R (*mm*^3^), Macula T Out R (*mm*^3^), Macula N Out R (*mm*^3^), Total macular (*mm*^3^)	Excluded	C-SD	GQ
[78]	MCI: 27, FTD: 17, HCs: 49, Mild AD: 20, Mod AD: 17	MCI: 70.45±5.51, Mild AD: 69.75±7.51, Mod AD: 71.23±6.95, FTD: 65.59±6.89, HCs: 68.32±6.96	CS	pRNFL and GCL-IPL thicknesses	Excluded	H-SD	GQ
[79]	FTD: 27, HCs: 44	FTD: 65.8±7.6, HCs: 55.9±11.9	CS	Total retina, outer retina, mRNFL, GCL, IPL, INL, OPL, ONL, EZ, POS, IZ, RPE	Excluded	H-SD	GQ
[80]	Initially: FTD: 27, HCs: 44 Follow-up: FTD: 16 (with subgroup of tauopathy: 9), HCs: 30	FTD: 65.6±8.2, tauopathy: 65.8±6.7, HCs: 53.1±11.3	L	Total retina, Outer retina, mRNFL, GCL, IPL, OPL, ONL, EZ, POS, IZ, RPE thicknesses	Excluded	H-SD	GQ
[81]	HCs: 172, *CH*_*NonAgen*_: 52, *CI*_*NonAgen*_: 23	HCs: 70.4±7.5, *CH*_*NonAgen*_: 92.4±1.9, *CI*_*NonAgen*_: 91.9±2.9	CS	Total MRT Inn R (μm), Total MRT Out R (μm), mRNFL Inn R (μm), mRNFL Out R (μm), GCL Inn R (μm), GCL Out R (μm), IPL Inn R (μm), IPL Out R (μm), pRNFL Avg, pRNFL NS, pRNFL N, pRNFL NI, pRNFL TI, pRNFL T, pRNFL TS, CRAE, CRVE, AVR, FDa, FDv, cTORTa, cTORTv	Excluded	H-SD	GQ
[82]	mild AD: 50, HCs: 152	mild AD: 73.10±5.36, HCs: 71.03±4.62	CS	pRNFL G, pRNFL TS, pRNFL T, pRNFL TI, pRNFL NI, pRNFL N, pRNFL NS. RT of 1-, 3- and 6-mm diameter centered at the fovea RT-S3, RT-T3, RT-I3, RT-N3, RT-S6, RT-T6, RT-I6, RT-N6.	Excluded	H-SD	GQ
[83]	Initial participants: 430 Drop-out group: 215, Follow-up group: 215	Initial participants: 76.3±6.6, Dropout group: 76.2±6.5, Follow-up: 80.9±6.5,	L	Outer, inner, total of GCL, IPL, INL, OPL, ONL, mRNFL, average of pRNFL thicknesses, and SFCT	Excluded	H-SD	GQ
[84]	MCI: 45, HCs: 104	MCI: 74.4±3.2, HCs: 74.1±2.6	CS	pRNFL Total, pRNFL S, pRNFL I, pRNFL N, pRNFL T	Excluded	C-HD	GQ
[85]	MCI due to AD: 24, HCs: 31 *APOE* ɛ4+:10, *APOE* ɛ4-: 18	MCI due to AD: 72.8±8.6, HCs: 69.0±10.4 *APOE* ɛ4+: 71.1±7.1, *APOE* ɛ4-: 73.2±6.5	CS	The thicknesses of the total MRT, mRNFL, GCL, IPL, INL, OPL, ONL, RPE. FAZ and VD of the SCP and DCP, as well as the SCP VD in the following subfields: Central, S Inn R, I Inn R, T Inn R, N Inn R, S Out R, I Out R, T Out R, N Out R.	Excluded	C-HD	GQ
[86]	AD Dementia:14, PD: 19, HCs: 31	AD Dementia: 64.7±9.7, PD: 62.9±9.7, HCs: 65.1±7.6	CS	ILM-RPE *mm*^3^, ILM-RPE μm, ONL-RPE *mm*^3^, ONL-RPE μm, ONL-EZ *mm*^3^, ONL-EZ μm, EZ-RPE *mm*^3^, EZ-RPE μm	Excluded	C-HD	GQ
[87]	Mild AD: 20, HCs: 28	AD: 79.3 + 4.1, HCs: 72.1 + 5.1	CS	Fovea (um), S Macula Inn R, I Macula Inn R, T Macula Inn R, N Macula Inn R, S Macula Out R, I Macula Out R, T Macula Out R, N Macula Out R, pRNFL S, pRNFL I, pRNFL T, pRNFL N, Total macular *mm*^3^	Excluded	T-3D	GQ
[88]	Mild AD: 17, HCs: 15	NA	CS	SFCT, ChorT at 500, 1000, and 1500 microns in the S, I, T and N quadrants.	Excluded	H-SD	LQ
[89]	AD: 324, MCI: 192, HCs: 414	AD: 78.99±7.87, MCI: 76.46±7.14, HCs: 65.93±9.01	CS	pRNFL thickness in quadrants: S, I, T, N	Excluded	T-3D	GQ
[90]	EOAD: 15, HCs: 15	EOAD: 62.20±63.67, HCs: 62.00±66.27	CS	Total MRT, Total MRV. Inn R, Out R, and foveal zone of MRT, mRNFL, GCL, IPL, INL, OPL, ONL, RPE	Excluded	H-SD	GQ
[91]	aMCI: 17, HCs: 17	aMCI: 74±NA HCs: 74±NA	CS	pRNFL Total, pRNFL S, pRNFL I, pRNFL N, pRNFL T, Macula volume (*mm*^3^), Macula GC-IPL (μm)	Excluded	H-SD	GQ
[26]	HCs: 145, AD: 77 (Mild AD: 31, Mod AD: 27, Severe AD: 19)	HCs: 60.34±7.14 AD: 61.94±8.41 (Mild AD: 60.91±7.47, Mod AD: 62.16±9.19, Severe AD: 63.33±8.92)	CS	VD of fovea, FAZ, Full Inn R, Full Out R. Inn R and Out R in quadrant S, I, T, N	Excluded	C-HD	GQ
[92]	AD: 159 versus HCs: 299	AD: 63.03±9.06 HCs: 61.55±8.92)	CS	mRNFL, GCL, IPL, INL, OPL, ONL, IS/OS, Outer Segment, OPR, RPE, and pRNFL thicknesses	Excluded	C-HD	GQ
[93]	AD: 6, PD: 6, HCs: 8	AD: 69±NA, PD: 74±NA, HCs: NA	CS	Macular cube volume; pRNFL thicknesses in S, I, T, N quadrants; and GCL is S, SN, IN, I, IT, ST.	Excluded	C-HD	GQ
[19]	CKD: 177, CKD_Low: 13, CKD_Middle: 65, CKD_High: 99	CKD: 64.7±6.6 CKD_Low: 67.8±7.4, CKD_Middle: 65.6±6.7, CKD_High: 63.7±6.3	CS	IVC FAZ area (*mm*^2^), Avg pRNFL (μm), GCC (μm), GCC-GLV%, GCC-FLV%, parafoveal VD of SVP and DVP.	Excluded	A-SD	GQ

**Abbreviations/Keywords (related to imaging machine type):** H-SD, Heidelberg SD-OCT; T-SS, DRI SS-OCT Triton; T-SD, Topcon SD-OCT; T-3D, 3D-OCT Maestro Topcon; D-Fundus, Dilated fundus examination; C-SD, Cirrus SD-OCT Carl Zeiss; C-HD, Cirrus HD-OCT; O-SD, Optos SD-OCT; A-SD, RTVue-XR Avanti SD-OCT; N-SD, Nidek RS-3000 Advance SD-OCT; N-Fundus, NIDEK AFC-330 to obtain fundus photography; N-Refract, NIDEK Tonoref II device to measure the refractive status and IOP; C-Refract, Autorefractor Canon RK-5; ℵ, no details about the machine. **Abbreviations/Keywords (related to cohort):** MCI, mild cognitive impairment; aMCI, amnestic type MCI; AD, Alzheimer’s disease; eAD, early AD; EOAD, amyloid-positive early-onset AD; mmAD, mild to moderate AD; HCs, healthy controls; *CH*_*NonAgen*_, cognitively healthy nonagenarians; *CI*_*NonAgen*_, cognitively impaired nonagenarians; PCA, posterior cortical atrophy; tAD, typical AD; PPG, pre-perimetric glaucoma; NC, normal cognition; PD, Parkinson’s disease; DLB, dementia with Lewy bodies; VCID, vascular cognitive impairment and dementia; FTD, frontotemporal dementia; E46K-SNCA, E46K mutation carriers in the α-synuclein gene; CKD, chronic kidney disease; CKD_Low, CKD Low MMSE scores; CKD_Middle CKD Middle MMSE scores; CKD_High, CKD High MMSE scores; MMSE, Mini-Mental State Examination. **Abbreviations/Keywords (extra details about cohort):** *M, the dementia group included AD, vascular dementia and DLB; *a, this group underwent RNFL thickness analysis; *b, this is a subsample group from *a but went for GCL, IPL, and INL volume measurements; *A+and *A- denote amyloid-proven (positive) and amyloid-negative, respectively; *BIOM*_+*ve*_ and *BIOM*_-*ve*_ indicate a positive and a negative amyloid measurement in CSF analysis, respectively. **Abbreviations/Keywords (general):** QA, quality assessment; std, standard deviation; NA, not available; IOP, intraocular pressure; AFI, adjusted flow index; G, Global; FMT, full macular thickness; CRT, central retinal thickness from a macular scan; Inn R, inner macular ring; Out R, outer macular ring; C, central; S, superior; I, inferior; T, temporal; N, nasal; ST, superotemporal; SN, superonasal; IT, inferotemporal; IN, inferonasal; NS, nasal superior; NI, nasal inferior; TS, temporal superior; TI) temporal inferior; Avg, average; RT, retinal thickness; RT S, RT superior; RT I, RT inferior; RT-SZ1, RT of S zone 1; RT-SZ *φ*, RT of S zone *φ*; RT-IZ1, RT of I zone 1; RT-IZ *φ*, RT of I zone *φ*; RT-*Δ*(1–5), the RT I subtracted from the corresponding RT S; GCL-*Δ*(1–5), the GCL I subtracted from the corresponding GCL S; FRT, full RT; MRT, macular retinal layer thickness; MRV, macular retinal volume; GCL, ganglion cell layer; IPL, inner plexiform layer; CG-IPL, ganglion cell-inner plexiform layer; GCC, ganglion cell complex; GCC-GLV%, GCC global loss volume; GCC-FLV%, GCC focal loss volume; INL, inner nuclear layer; OPL-ONL, outer plexiform-Henle fiber-outer nuclear layer; ELM, external limiting membrane; ELM-IS/OS, external limiting membrane - photoreceptor inner and outer segments; ILM, inner limiting membrane; OLM, outer limiting membrane; PhotoR, photoreceptors; OPR, outer segment PhotoR/RPE complex; NFL, nerve fiber layer; RNFL, retinal nerve fiber layer; mRNFL, macular retinal nerve fiber layer; pRNFL, peripapillary retinal nerve fiber layer; cpRNFL, circumpapillary retinal nerve fiber layer; CST, central subfield thickness; ChorT, choroidal thickness; SFCT, subfoveal ChorT; SCP, superficial capillary plexuses; DCP, deep capillary plexuses; CC, choriocapillaris; SVP, superficial vascular plexus; DVP, deep vascular plexus; VPD, vascular perfusion density; FD, fractural dimension; FDa, FD of arteries; FDv, FD of veins; CRAE, Central Retinal Artery Equivalent; CRVE, Central Retinal Vein Equivalent; AVR, Arteriole-Venular Ratio; cTORT, curvature Tortuosity; cTORTa, cTORT of arteries; cTORTv, cTORT of veins; CS, cross-sectional studies; L, longitudinal studies; AMB , ambiguous with no specific pRNFL or mRNFL.

**Table 3:** Cross-Sectional pRNFL related parameters

	pRNFL- Tot (μm)	pRNFL-G (μm)	pRNFL-Avg (μm)	pRNFL-S (μm)	pRNFL-I (μm)	pRNFL-T (μm)	pRNFL-N (μm)	pRNFL-TS (μm)	pRNFL-TI (μm)	pRNFL-NS (μm)	pRNFL-NI (μm)
**AD**
**AD vs. HCs:**
[77] AD: 21 vs. HCs: 25	–	–	–*^*NS*^*	–*^*NS*^*	–*^*NS*^*	–*^*NS*^*	–*^*NS*^*	–	–	–	–
[92] AD: 159 vs. HCs: 299	–	–	↓	↓	↓	–*^*NS*^*	–*^*NS*^*	–	–	–	–
[53] AD: 43 vs. HCs: 57	–	↓	–	↓	–*^*NS*^*	–*^*NS*^*	–*^*NS*^*	–	–	–	–
[17] AD: 42 vs. HCs: 45	–	–	↓	↓	↓	–	–	–	–	–	–
[93] AD: 6 vs. HCs: 8	–	–	–	↓	–*^*NS*^*	–*^*NS*^*	–*^*NS*^*	–	–	–	–
[69] AD: 150, HCs: 75	–	–	↓	–	–	↓	–*^*NS*^*	↓	↓	–*^*NS*^*	–*^*NS*^*
[64] AD: 17 vs. HCs: 22	–	–	↓	↓	↓	–*^*NS*^*	↓	–	–	–	–
[56] tAD: 23 vs. HCs: 70	–	–	–*^*NS*^*	–	–	–	–	–	–	–	–
**Mild/Early AD vs. HCs:**
[78] Mild AD: 20 vs. HCs: 49	–	–*^*NS*^*	–	–	–	–	–	–	–	–	–
[32] Mild AD: 37, HCs: 29	↓	–	–	–	–	–	–	–	–	–	–
[54] eAD: 40 vs. HCs: 40	–	–	↓	↓	–*^*NS*^*	–*^*NS*^*	–*^*NS*^*	–	–	–	–
[82] mild AD: 50 vs. HCs: 152	–	↓	–	–	–	↓	↓	↓	↓	↓	↓
[92] Mild AD: 51 vs. HCs: 299	–	–	–*^*NS*^*	–*^*NS*^*	–*^*NS*^*	–*^*NS*^*	–*^*NS*^*	–	–	–	–
[87] mild AD: 20 vs. HCs: 28	–	–	–	–*^*NS*^*	–*^*NS*^*	–*^*NS*^*	–*^*NS*^*	–	–	–	–
**Moderate/Severe AD vs. HCs:**
[78] Mod AD: 17 vs. HCs: 49	–	↓	–	–	–	–	–	–	–	–	–
[92] Moderate AD: 67, vs. HCs: 299	–	–	↓	↓	–*^*NS*^*	–*^*NS*^*	–*^*NS*^*	–	–	–	–
[92] Severe AD: 41 vs. HCs: 299	–	–	↓	↓	↓	↓	↓	–	–	–	–
**Mild moderate AD:**
[61] mmAD: 56 vs. HCs 56	–	–	↓	↓	↓	–*^*NS*^*	↓	–	–	–	–
[55] mmAD: 20 vs. HCs: 21	–*^*NS*^*	–	–	–*^*NS*^*	–*^*NS*^*	–*^*NS*^*	–*^*NS*^*	–	–	–	–
**MCI**
**MCI vs. HCs:**
[65] aMCI: 23 vs. HCs: 24	–	–	–*^*NS*^*	–*^*NS*^*	–*^*NS*^*	–*^*NS*^*	–*^*NS*^*	–	–	–	–
[84] MCI: 45 vs. HCs: 104	–*^*NS*^*	–	–	–*^*NS*^*	–*^*NS*^*	–*^*NS*^*	–*^*NS*^*	–	–	–	–
[91] aMCI: 17 vs. HCs: 17	–*^*NS*^*	–	–	–*^*NS*^*	–*^*NS*^*	–*^*NS*^*	–*^*NS*^*	–	–	–	–
[33] aMCI: 54 vs. HCs: 54	–	–	↓	↓	↓	–	–	–	–	–	–
[53] MCI: 37 vs. HCs: 57	–	↓	–	↓	–*^*NS*^*	–*^*NS*^*	–*^*NS*^*	–	–	–	–
[17] MCI: 48 vs. HCs: 45	–	–	↓	↓	↓	–	–	–	–	–	–
[78] MCI: 29 vs. HCs: 49	–	↓	–	–	–	–	–	–	–	–	–
[63] non-amnestic MCI: 17 vs. HCs: 56	–	–	–*^*NS*^*	–	–	–	–	–	–	–	–
[63] amnestic MCI: 59 vs. HCs: 56	–	–	–*^*NS*^*	–	–	–	–	–	–	–	–
[81] *CI*_*NonAgen*_: 23 vs. HCs: 172	–	–	↓	–	–	–*^*NS*^*	–*^*NS*^*	↓	↓	–*^*NS*^*	–*^*NS*^*
[19] CKD_Low: 13 vs. CKD_High: 99	–	–	–*^*NS*^*	–	–	–	–	–	–	–	–
**Multi-Comparisons:**
[50] MCI: 324 vs. Dementia: 38 vs. HCs: 613	–	–*^*NS*^*	–	–*^*NS*^*	–*^*NS*^*	–*^*NS*^*	–*^*NS*^*	–	–	–	–
[72] MCI: 66 vs. Dementia: 43 vs. HCs: 27	–	–	–*^*NS*^*	–	–	–	–	–	–	–	-
[72] MCI: 66 vs. AD: 17 vs. DLB: 16 vs. VCID: 6 vs. FTD: 4 vs. HCs: 27	–	–	–*^*NS*^*	–	–	–	–	–	–	–	–
[63] amnestic MCI: 59 vs. non-amnestic MCI: 17 vs. HCs: 56	–	–	–*^*NS*^*	-	–	–	–	–	–	–	–
[81] *CH*_*NonAgen*_: 52 vs. *CI*_*NonAgen*_: 23 vs. HCs: 172	–	–	–*^*NS*^*	–	–	–*^*NS*^*	–*^*NS*^*	–*^*NS*^*	–*^*NS*^*	–*^*NS*^*	–*^*NS*^*
[62] MCI: 24 and AD: 23 vs. HCs: 43	–	↓	–	–	–	↓	–*^*NS*^*	↓	↓	–*^*NS*^*	–*^*NS*^*
[66] MCI: 15, mmAD: 15, HCs: 18	–	–*^*NS*^*	–	–	–	–*^*NS*^*	–*^*NS*^*	–*^*NS*^*	–*^*NS*^*	–*^*NS*^*	–*^*NS*^*
[89] AD: 324, MCI: 192, HCs: 414	–	–	–	–*^*NS*^*	–*^*NS*^*	–*^*NS*^*	–*^*NS*^*	–	–	–	–
**Special Comparison**
[90] EOAD: 15, HCs: 15	–	–*^*NS*^*	–	–	–	–*^*NS*^*	–*^*NS*^*	–*^*NS*^*	–*^*NS*^*	–*^*NS*^*	–*^*NS*^*
[75] CH-PAT: 27 vs. CH-NAT: 16	–	↓	–	–	–	–	–	–	–	–	–
[78] FTD: 17 vs. HCs: 49	–	↓	–	–	–	–	–	–	–	–	–
[78] FTD: 17 vs. Mild AD: 20	–	↓	–	–	–	–	–	–	–	–	–
[92] Severe AD: 41 vs. Mild AD: 51	–	–	↓	–*^*NS*^*	–*^*NS*^*	–*^*NS*^*	↓	–	–	–	–
[81] *CH*_*NonAgen*_: 52 vs. HCs: 172	–	–	↓	–	–	–*^*NS*^*	↓	↓	↓	↓	↓
[63] aMCI: 59 vs. non-amnestic MCI: 17	–	–	–*^*NS*^*	–	–	–	–	–	–	–	–
[78] Moderate AD: 17 vs. Mild AD: 20	–	–*^*NS*^*	–	–	–	–	–	–	–	–	–

See Table 2 for definitions of abbreviations.

**Table 4:** Longitudinal pRNFL related parameters

	pRNF L-Tot (μm)	pRNF L-G (μm)	pRNF L-Avg (μm)	pRNF L-S (μm)	pRNF L-I (μm)	pRNF L-T (μm)	pRNF L-N (μm)	pRNF L-TS (μm)	pRNF L-TI (μm)	pRNF L-NS (μm)	pRNF L-NI (μm)	pRNFL -Tot Δ*	pRNF L-S Δ*	pRNF L-I Δ*	pRNF L-T Δ*	pRNF L-N Δ*
[51] Mild to moderate AD: 36 vs. HCs: 36 (12 month)	–	–	–	–	–	–	–	–	–	–	–	↓	↓	↓	–*^*NS*^*	–*^*NS*^*
[70] *Stable* _ *Total*: 60 vs. *Converted* _ *Total*: 18	–	–*^*NS*^*	–	–*^*NS*^*	↓	–*^*NS*^*	–*^*NS*^*	–	–	–	–	–	–	–	–	–
[35] aMCI: 19 (2 years follow-up vs. baseline)	–	–	↓	–	–	–	–	–	–	–	–	–	–	–	–	–
[52] *MCI*_*AD*_: 9 vs. *MCI*_*MCI*_: 12
(follow-up 2 years)	–	–	–*^*NS*^*	–*^*NS*^*	–*^*NS*^*	↓	–*^*NS*^*	–	–	–	–	–	–	–	–	–

The quality assessment results of the included research papers’ based on [9] were documented in Table 2. An example of a HQ assessed work would be the study by [53], since the diagnosis of AD was supported by Aβ_42_, t-tau, and p-tau and the study was well organized and descriptive. On the contrary, [73] can be considered as LQ work because it failed to provide sufficient information about whether this RNFL thickness is around the optic disc or around the fovea. Nevertheless, [50] failed to distinguish dementia subtypes since patients with AD, vascular dementia (VaD), and dementia with Lewy bodies (DLB) were included in the same dementia group and hence, the quality assessment for this paper was LQ. Given that there are some discrepancies among various dementia subtypes, as discussed earlier in the review, this is a considerate drawback of the study [50]. Another study by [48] included dementia, AD, and VaD patients; however, the exact diagnosis was not clearly mentioned which is a limitation to this study. In addition, they study by [48] failed to provide the details of pRNFL thickness such that it could be an average or global pRNFL thickness, and hence inconsistence with the current review and difficult in comparison with other research works. Another limitation was observed in [34] due to not using capillaries for the vascular density of the retina, and hence, the results could potentially be improved if these small capillaries are utilized and included in the analysis.

The included studies in this review excluded all eye diseases like glaucoma and age-related macular degeneration. The only exception studies are by [33] which included pre-perimetric glaucoma (PPG) patients, as well as the study by [34] which did not include the eye health status of the cohort’s individuals.

### Cross-sectional results for structural parameters

#### Optic disc related parameters

The peripapillary RNFL (pRNFL), which is around the optic disc, measurements could be mainly split into total pRNFL (pRNFL-Tot), global pRNFL (pRNFL-G), and average pRNFL (pRNFL-Avg) [94]. The pRNFL-Tot represents the total thickness summation of all six sectors around the optic disc (T, TS, TI, N, NS, NI), pRNFL-G considers the thickness of the entire pRNFL circumference around the optic nerve head (ONH), and finally pRNFL-Avg which incorporates the averaged pRNFL thickness over a circular area around the ONH [94]. The pRNFL-Tot thickness was studied [32, 55, 84, 91] to differentiate between cognitively impaired (CI) and healthy participant (HCs). Only in [32], the pRNFL-Tot thickness was significantly thinner for mild Alzheimer’s (mild AD) when compared to HCs. Conversely, pRNFL-Tot showed insignificant thickness variations between mild-to-moderate AD (mmAD) compared to HCs in [55], and MCI against HCs in [84, 91].

Nevertheless, the pRNFL-G thickness was explored by [16, 50, 53, 62, 66, 75, 78, 82, 90]. A significant thinning was revealed when comparing AD in [53], mild AD in [82], and moderate AD in [78] against HCs. In addition, a notable decrease in pRNFL-G thickness was found for MCI groups compared to HCs in [53, 78] and for a combined MCI/AD group in contrast to HCs in [62]. Moreover, the frontotemporal dementia (FTD) group in [78] had a notable pRNFL-G thickness thinning when compared independently against HCs and against mild AD. Another interesting research by [75] aimed to detect CSF Aβ_42_, tau, and Aβ_42_/tau ratio before cognitive decline with the aid of retinal thickness extracted by OCT. The study by Asanad et al. [75] involved two cognitively healthy (CH) groups one with Normal Aβ_42_/tau ratio (CH-NAT), and another group with pathological Aβ_42_/tau ratio (CH-PAT). The pRNFL-G thickness was statistically significantly thinner for CH-PAT group contrary to CH-NAT group. On the contrary, pRNFL-G thickness was unsuccessful in discriminating between EOAD in [90], mild AD in [78], and a combined aMCI/AD group in [16] with respect to HCs. Additionally, pRNFL-G thickness was ineffective when multiple assessments were conducted in [50, 66]. Simply, the thickness variations of pRNFL-G were insignificant when comparing multiple groups (MCI, dementia, and HCs) in [50] as well as when contrasting other groups with varied degrees of cognitive impairments (MCI, mmAD, and HCs) in [66]. Furthermore, the pRNFL-G thickness changes were negligible when comparing moderate AD and mild AD subgroups as well as moderate AD against MCI group in [78].

The pRNFL-Avg thickness was studied in [17, 19, 33, 54, 56, 61, 63–65, 69, 72, 77, 81, 92] in an attempt to distinguish between CI, AD, MCI, DLB, vascular cognitive impairment and dementia (VCID), FTD patients, and HCs. A noteworthy thickness decline of pRNFL-Avg was found when comparing early AD (eAD) in [54], mmAD in [61], AD in [17, 64, 69, 92], moderate AD and severe AD in [92] in contrast to HCs. Likewise, a significant thinning of pRNFL-Avg was reported in [17, 33] for MCI and aMCI as opposed to HCs. The study by [92] also documented a noticeable pRNFL-Avg thickness thinning in severe AD in comparison with mild AD, and hence AD progression was identified by this retinal biomarker. On the other hand, the pRNFL-Avg thickness was ineffective in drawing a distinction between AD in [77], mild AD in [92], typical AD (tAD) in [56], aMCI in [65], non-amnestic MCI and aMCI in [63], and HCs. Also, aMCI and non-amnestic MCI groups in [63] had a similar pRNFL-Avg thickness variations, and negligible differences. Furthermore, another study by [19] which involved analyzing the cognitive functions of chronic kidney disease (CKD) patients and the cohort was split based on Mini-Mental State Examination (MMSE) score into three groups: CKD_Low (MMSE < 24), CKD_Middle (24≤ MMSE ≤ 27), and CKD_High (MMSE > 27). [19] demonstrated a considerable pRNFL-Avg thickness reduction for CKD_High group with respect to CKD_Low. Nevertheless, pRNFL-Avg thickness was ineffective when realizing multiple comparisons in [63, 72, 81]. Specifically, the pRNFL-Avg thickness variations in [72] were insignificant when comparing MCI, dementia, and HCs in [72], and when correlating MCI, AD, DLB, VCID, FTD, and HCs. Besides, the pRNFL-Avg thickness also failed to separate between aMCI, non-amnestic MCI, and HCs in [63], and between *CH*_*NonAgen*_, *CI*_*NonAgen*_, and HCs in [81].

The pRNFL thickness was also studied in various main quadrants (S, I, T, N) in [17, 33, 50, 53–55, 61, 62, 64–66, 69, 77, 81, 82, 84, 87, 89–93] and in special sectors (TS, TI, NS, NI) in [62, 66, 69, 81, 82, 90]. The pRNFL-S thickness was significantly decreased for AD in [17, 53, 64, 92, 93], for eAD in [54], moderate AD and severe AD in [92], mmAD in [55, 61], MCI in [17, 53], and aMCI in [33] when compared to HCs. A noticeable thinning in pRNFL-I thickness was found in [17, 64, 92] for AD, in [61] for mmAD, in [33] for aMCI, in [17] for MCI, and in [92] for severe AD when contrasted with HCs. Another distinguishable thinning in pRNFL-T thickness was reported for AD in [69], mild AD in [82], Severe AD in [92], and MCI in [62] in comparison to HCs. Likewise, a distinct thinning for pRNFL-N was observed for AD in [64], mild AD in [82], Severe AD in [92], and mmAD in [61] as opposed to HCs. Contrariwise, the thickness variations of pRNFL in four quadrants (S, I, T, N) were negligible between AD in [77], mild AD in [87, 92], mmAD in [55], MCI in [84], and aMCI in [65, 91] when contrasted with HCs. Another interesting comparison was conducted in [92] such that only pRNFL-N was significantly thinner in severe AD compared to mild AD, whilst changes in other quadrants of pRNFL were minor. All four quadrant of pRNFL (except for T) showed a significant thinning for AD patients in [64] and mmAD in [61] as opposed to HCs group. Slightly contradicting results in [53, 93] where only the pRNFL-S thickness changes were considerable, whilst thickness changes in other pRNFL quadrants were negligible when comparing AD against HCs. However, all studied by [53, 64, 93] agree that pRNFL-S is indeed able to distinguish between AD and HCs. Similarly, the study by [54] also comply with the findings of [53, 64, 93] such that only the pRNFL-S thickness was reduced for eAD as opposed to HCs, whereas thickness variations in other quadrants were minor. Moreover, except for pRNFL-S, the thicknesses of other quadrants of pRNFL were unsuccessful in differentiating moderate AD against HCs in [92]. On the other hand, all pRNFL quadrants (S, I, T, N) thicknesses were attenuated for severe AD with respect to HCs in [92]. Unexpectedly, only the pRNFL-S was prominently thinner for MCI group compared to HCs in [53], while pRNFL variations in other quadrants’ thicknesses were insignificant. The pRNFL thicknesses in TS, TI, NS, and NI sectors were all prominently thinner for mild AD in [82], whereas only TS and TI sectors were notably thinner for AD in [69] compared with HCs. Only a contradicting study by [77] failed to report any significant pRNFL-S thickness changes between AD and HCs groups. In fact, all four quadrants of pRNFL thickness variations (S, I, T, N) were minor between AD and HCs in [77]. Moreover, compared to HCs, pRNFL-S thickness changes were negligible for mild AD in [87, 92] and mmAD in [55]. Indeed, the pRNFL thickness alterations in S, I, T, and N quadrants were insignificant in [55, 87, 92]. On the contrary, only pRNFL-T thickness variations were negligible while other quadrants (S, I, N) had a significant thinning for mmAD in [61] with respect to HCs. When analyzing pRNFL-I thickness changes, a discrepant outcome is documented such that notable thinning was associated with AD patients in [64, 92] compared with HCs, while insignificant thinning was concluded between AD and HCs in [53, 93]. When pRNFL thickness was analyzed in both T and N sectors, studies by [53, 92, 93] yielded no relevant thickness differences between AD patients and HCs. Moreover, inconsequential results are revealed when comparing the pRNFL thickness (T, N) for AD and HCs, such that the variations of pRNFL-T in [64] and pRNFL-N in [69] were insignificant; however, notable thinning of pRNFL-T and pRNFL-N was indicated for AD in [69] and [64], respectively. The findings from study [54] suggest that only pRNFL-S had a notable thinning for eAD compared to HCs, while other pRNFL thickness changes in I, T, and N sectors were insignificant. Other research works failed to utilize pRNFL thickness to differentiate MCI in [84] and aMCI in [65, 91] individually against HCs, where thickness variations in four quadrants (S, I, T, N) were negligible. Only one study by [53] reported a prominent thinning in pRNFL-S for MCI patients with respect to HCs, whilst other variations of pRNFL thicknesses in I, T, and N quadrant were insignificant. A multi-class comparison was performed by [62] such that a significant pRNFL thickness thinning in T, TS, and TI sectors was found between AD, MCI, and HCs, whilst a negligible pRNFL thickness alteration in N, NS, and NI sections. Nevertheless, other multiple groups comparisons based on four quadrants of pRNFL thicknesses also deemed to failure in [50] for MCI, demented, HCs people as well as in [89] between AD, MCI, and HCs individuals. Moreover, other insignificant pRNFL thickness changes in T, N, TS, TI, NS, NI quadrants was documented in [66] between MCI, mmAD, and HCs.

An interesting study by [90] failed to correlate pRNFL thickness variations in T, N, TS, TI, NS, and NI sectors between amyloid-positive early-onset AD (EOAD) and HCs. Moreover, another special study by [81] involved Healthy nonagenarians (*CH*_*NonAgen*_), cognitively impaired (CI) nonagenarians (*CI*_*NonAgen*_), and HCs where nonagenarians are people aged between 90–100. A prominent decrease in pRNFL-Avg, pRNFL-TS, pRNFL-TI thicknesses were associated with *CI*_*NonAgen*_ contrasted with HCs in [81]. Contrariwise, the pRNFL thickness changes in sectors (T, N, NS, NI) were negligible for *CI*_*NonAgen*_ with respect to HCs. Surprisingly, in [81], *CH*_*NonAgen*_ group also had a significantly thinner pRNFL-Avg, pRNFL-N, pRNFL-TS, pRNFL-TI, pRNFL-NS, and pRNFL-NI thicknesses compared with HCs. Contrarily, only pRNFL-T thickness variations were insignificant between *CH*_*NonAgen*_ and HCs. When comparing the three groups (*H*_*NonAgen*_, *CI*_*NonAgen*_, and HCs) in [81], all the pRNFL parameters where insignificant. Compared to HCs in [32], the mild AD group had a significantly thinner pRNFL in GH S-Hemi, GH I-Hemi, GH-SN, GH-NS, GH-NI, and GH-IN sections compared to HCs, whilst other negligible pRNFL thickness variations were found in GH-TS, GH-ST, GH-IT, and GH-TI between the groups.

### Macula-related parameters

Changes to the full retinal thickness (FRT), defined from the ILM to the top of RPE, failed to distinguish between tAD in [56], AD in [69], mild AD in [42, 47], MCI in [85], aMCI in [65], EOAD in [90], FTD in [79, 80], against HCs. Additionally, when multiple classes were involved in the analysis, the FRT variations were negligible between the three groups (mild AD, moderate AD, HCs) in [76], and between (aMCI, AD, HCs) in [67]. According to [69, 92], AD patients had a notable thinning of the GCL and IPL in contrast to HCs. In addition, in comparison to HCs, a substantial thickness reduction of GC-IPL in [25] and GCC in [17] for AD group. Moreover, both moderate AD and severe AD groups individually had a prominent thinning of GCL and IPL in [92] compared with HCs. Furthermore, a notable thickness reduction of GCL and IPL volumes was associated with AD patients unlike HCs [53]. Moreover, the outcomes of [69] indicated a significant thinning of macular RNFL (mRNFL), GCL, IPL, and ONL for AD contrasted with HCs. The results in [42] align with those in [47], indicating that a pronounced thinning of macular layers mRNFL, INL, and OSL is related to mild AD rather than to HCs. Although both works by [92] and [42] agreed that a noteworthy thinning of GCL and IPL layers is associated with mild AD unlike HCs; however, the findings of [47] is conflicting such that the thickness changes of both GCL and IPL layers were insignificant between both groups. The results reported in [17] agreed with [33] such that a notable thickness reduction of GCC layer is associated with MCI or aMCI rather than HCs. The analysis of performed by [72] indicated that the GC-IPL thickness is biomarker which is able to differentiate between three groups (MCI, dementia, HCs) as well as dementia subgroups (AD, DLB, VCID, FTD) against MCI and HCs in [72]. Additionally, changes in macula volume was significantly different among groups (AD, DLB, VCID, FTD, MCI, HCs) in [72]. When comparing the finding of [79] and [80], a majorly thinner ONL and IS-OS, also referred to as EZ, was associated with FTD and tauopathy groups in [80] as well as FTD, unknown pathology, and probable tauopathy in [79] all individually compared against HCs. Another interesting biomarker is OPL thickness changes, where the FTD groups in [79] and [80] had a significant OPL thinning compared to their corresponding HCs. The thickness of GC-IPL was substantially reduced for moderate AD and FTD with respect to HCs in [78]. Interestingly, the GC-IPL thickness variations was an excellent biomarker to detect the progression of neurodegenerative disorders in [78]. For instance, the GC-IPL thickness was considerably decreased when comparing FTD versus mild AD, moderate AD versus MCI, and moderate AD versus mild AD [78]. Other studies supported by CSF and/or brain MRI also attempted to investigate the significance of FRT changes, such that only tauopathy group had a notable FRT thinning compared with HCs; however, when adjusting the linear models, the FRT parameter was not significant anymore. The other supported studies demonstrated minor FRT changes when comparing TDP-43 and probable tauopathy groups in [79] individually against HCs.

There were other studied retinal parameters in the literature; however, some of these parameters are only associated with a single research work. For instance, significant changes to the Minimal GCL+IPL layer was an indicator to differentiate between 3 groups (MCI, Dementia, HCs) and 6 groups (MCI, AD, DLB, VCID, FTD, HCs) in [72]. In [87], the foveal macular thickness (MT), inner ring of MT in S, I, T, N quadrants, and only the T sector of the MT outer ring were notably reduced for mild AD comparatively with HCs. [76] studied the total retinal thickness (RT), total GCL thickness, RT and GCL thicknesses in S and I quadrants, as well as splitting the macula into S and I hemispheres with 5 zones each. The 5 RT zones in the S hemisphere were RT-SZ1, RT-SZ2, RT-SZ3, RT-SZ4, and RT-SZ5, whereas in the I hemispheres were RT-IZ1, RT-IZ2, RT-IZ3, RT-IZ4 and RT-IZ5. Additionally, RT-*Δ*1, RT-*Δ*2, RT-*Δ*3, RT-*Δ*4 and RT-*Δ*5 were all calculated by subtracting the mean inferior RT values from their corresponding mean superior RT. The previous procedure was repeated for GCL creating 5 superior zones: GCL-SZ1, GCL-SZ2, GCL-SZ3, GCL-SZ4, GCL-SZ5; 5 inferior zones: GCL-IZ1, GCL-IZ2, GCL-IZ3, GCL-IZ4, GCL-IZ5; and 5 differences (*Δ*): GCL-*Δ*1, GCL-*Δ*2, GCL-*Δ*3, GCL-*Δ*4, GCL-*Δ*5. There was no noticeable disparity between the three groups in terms of total RT and GCL thicknesses as well as RT and GCL thicknesses in S and I quadrants. Conversely, a discernible difference was found between groups, particularly in GCL-SZ2 and GCL-IZ2 thicknesses. Simply, the thickness of GCL-SZ2 was 41.26±6.95, 35.73±6.97, and 42.71±6.9 (*p* = 0.025) for Mild AD, Mod AD, and HCs, respectively. Moreover, the thickness of GCL-IZ2 was GCL-IZ2 39.87±6.36, 36.51±6.42, and 42.16±6.2 (*p* = 0.048) for Mild AD, Mod AD, and HCs, respectively. The rest of the parameters were insignificant between the groups. Garway-Heath (GH) map introduced in [31], shown in Fig. 5, was implemented by [32] to include two splitting patterns (quadrants) around the optic disc as well as around the fovea. The OCT optic disc parameters were comprised of the overall pRNFL thickness and pRNFL following GH in S & I Hemisphere (Hemi) and GH in 8 sections illustrated in Fig. 5.-a. Besides, the OCTA optic disc parameters were the VD of RPC (whole and peripapillary region), RPC VD pursuing GH in S & I Hemi and GH in 8 sections. On the other hand, the OCT foveal parameters were whole foveal thickness, macular thickness four quadrants (S, I, T, N), and macular thickness adopting GH in S & I Hemi method. Additionally, the OCTA foveal parameters contained the VD of SCP and DCP each evaluated at whole fovea, total parafovea, parafovea S & I hemi method, and parafovea in four quadrants. Only TI, IT, TS, and ST GH sections of pRNFL thicknesses were insignificant between the groups; however, all other OCT optic disc parameters were significantly thinner for AD group compared to HCs (*p* < 0.05). Additionally, all OCTA optic disc parameters (except VD of RPC GH-IT *p* = 0.066) were statistically significant between the groups. Oppositely, the OCT macular thickness parameters failed to distinguish between the groups (*p* > 0.05); however, the OCTA VD of macular SCP (I-Hemi: *p* = 0.026, T: *p* = 0.007, N: *p* = 0.003), and DCP (I-Hemi: *p* = 0.049, T: *p* = 0.013) were significantly reduced in mild AD patients compared to HCs. These OCT/OCTA significant changes indicates a damage to both the retinal microvascular system and retina neurons which is linked with cognitive decline. [32] correlated VD of the retina with memory, overall cognition, and visual-spatial perception Functions. The SN sector of peripapillary RPC VD can predict overall cognitive, executive, and visuospatial functions. Additionally, the NS and TS sectors of peripapillary RPC VD can predict memory function. Furthermore, the NI sector of peripapillary RPC VD can predict visuospatial function. Moreover, the T and S-Hemi sectors of macular SCP VD can predict executive function, while the T sectors of macular DCP VD can predict executive function. The reduced retinal VD is associated to AD; however, the novelty of [32] is the particularity in showcasing exactly which areas of the retina are VD reduced.

### Cross-sectional results for vascular parameters

#### Macula and optic disc related parameters

In the literature, negligible FAZ area changes was documented in the literature [16, 18, 22, 63, 71]; however, the FAZ Area was considered a reliable biomarker for cognitive impairment in other findings by [20, 21, 60, 85]. The FAZ area in the IVC was enlarged for biomarker positive group (*BIOM*_+*ve*_) compared to biomarker negative group (*BIOM*_-*ve*_) in [60]. Additionally, Alzheimer’s type dementia ATD group had a bigger FAZ area in the IVC with respect to HCs [20]. Compared to HCs, the MCI group had a larger FAZ area in the DCP [85]. Additionally, the FAZ area in IVC was statistically significantly different when comparing the three groups of MCI, AD, and HCs [21], where AD group had the biggest FAZ area. Strangely, even though both studies [16, 21] used the same A-SD machine, the results of [21] where opposing to [16]. Simply, the FAZ area changes in the IVC failed to discriminate between aMCI/eAD and HCs groups in [16]. Another difficulty to investigate the FAZ biomarker is the use of different OCT machines. For instance, the comparison between FAZ biomarker in works by [71] and [26] is not fair, simply because [71] and [26] works used H-SD and C-HD, respectively.

The VD biomarker for DCP and SCP were studied by [21], the findings of [21] showed that AD patients had a significant microvascular loss with respect to HCs in all parafovea (inner ring) and perifovea (outer ring) sectors of the DCP (*p* < 0.001), and only in SI section of the SCP. Moving on to MCI patients, where a significant microvascular loss in all parafovea sectors of DCP (except for NI), and only the SE of the perifovea of the DCP was notable (*p* < 0.001). Hence, VD changes captured mainly at all sectors of DCP could be used as a biomarker to detect AD; however, the detectability power of DCP-VD is limited to certain sectors. The DCP-VD was also found to be significantly lower in both *APOE* ɛ4 + and AD groups, who used the same C-HD OCT machine, when compared to their respective controls, *APOE* ɛ4- and HCs in [85] and [24], respectively. Conversely, SCP-VD changes did not differ significantly between *APOE* ɛ4 + and *APOE* ɛ4- groups in [85], but were found to be significantly lower in the AD group compared to HCs in [24]. Study by [25] reports conflicting results with studies [85] and [24], as it found that changes in both SCP-VD and DCP-VD parameters were not statistically significant when comparing AD and HCs. However, it should be noted that the sample size in [25] was relatively small, consisting of only 7 AD patients. Both studies [22] and [85] attempted to utilize both SCP-VD and DCP-VD parameters to differentiate between MCI and HCs. However, study [22] reported significant variations in DCP-VD but negligible changes in SCP-VD, while study [85] found noteworthy variations in SCP-VD but minor changes in DCP-VD. The ICP-VD was prominently reduced for MCI due to AD group compared to HCs in [22]; however, no other works in the literature investigated this biomarker with such special layers definition indicated by Table 1., and hence the reliability of ICP-VD is doubtful. Another study by [20] found that SVP-VD was significantly decreased in ATD compared to HCs; however, the SVP definition by [20] shown in Table 1. was only replicated in [19]. Additionally, the study by [19] involved cognitive impairments patients not ATD, and hence, not enough evidence to support SVP-VD biomarker.

According to the study [22], there was a noteworthy decrease in VLD at SCP, DCP, and ICP when comparing individuals with MCI to HCs. While studies [24] and [22] reported successful discrimination between MCI and HCs using SCP-VLD parameter, the trustworthiness of this parameter is questionable as study [16] failed to differentiate between aMCI and HCs.

Furthermore, in [32], both the VD of the peripapillary RPC (pRPC-VD) and the entire image of RPC (RPC-VD W-Img) were analyzed, and it was discovered that they were significantly lower in individuals with mild AD as compared to HCs. However, there is a lack of prior research on these biomarkers (pRPC-VD and RPC-VD W-Img) in the literature, which raises concerns about its effectiveness.

The FD, another parameter that studies the vascular complexity, was utilized to differentiate between AD and MCI compared to HCs, and it was deemed successful [24]. The results in [24] showed that the SCP-FD was significantly reduced in both the AD and MCI groups when compared individually against HCs. However, no significant differences were observed for DCP-FD changes between the groups. Another study by [22] also document a significantly higher FD for MCI due to AD group compared to HCs. However, H-SD OCT machine used in [22] which is different to the C-HD used in [24]. Additionally, the FD association was not clearly defined in [22], and hence, know known if the FD belongs to SCP or DCP layers. Therefore, the FD shows a good potential as a biomarker for cognitive decline; however, further research with different cohorts must be investigated in the future works.

### Longitudinal results for both structural and vascular parameters

Interestingly, the total pRNFL thinning rate for AD patients (over 12 months) was –1.6±2.3μm which is greater than the –0.6±1.4μm (p-value=0.04) in [51] and the average of 0.5μm associated with natural thinning due to aging [95]. The pRNFL thinning rate in the S section was –2.1±3.0 and –0.5±3.3 for AD and HCs (p-value=0.01), respectively. Moreover, the pRNFL thinning rate in the I section was –2.9±3.2 for AD patients which is far greater than the thinning rate of –0.6±2.5 for HCs (p-value=0.001), respectively. Conversely, the thinning rate of pRNFL in T and N quadrants were negligible. Hence, [51] concludes that a higher thinning rate of pRNFL (S, I, total) over longitudinal visits may indicate cognitive decline. Another longitudinal study by [70] which analyzed pRNFL thickness changes over the course of 25 month. The thickness of global pRNFL as well as the thicknesses in S, I, T, and N quadrants were assessed for 20 MCI patients and 58 HCs initially. Later in the study, 9 MCI patients converted to Mild-AD, 1 MCI converted to Moderate-AD, and 10 MCI patients remained stable. Additionally, 8 HCs converted to MCI and 50 HCs remained stable, and hence, there is a total of 60 and 18 stable and converted participants, respectively. This study focused on comparing stable and converted groups and only the pRNFL thickness in the I sector was significantly reduced between both groups. In fact, the converted group had an pRNFL I thickness of –11.0±12.8 μm whereas the stable group had a thickness of 0.4±15.7 μm (*p* = 0.009). The results by [70] imply that patients with a higher thickness reduction of pRNFL in the I sector could potentially be more susceptible to switching to a worse cognitive state, such as from MCI to AD dementia.

In terms of structural layer thickness, a longitudinal study by [83] evaluated the GCL, IPL, INL, OPL, ONL, mRNFL thicknesses (outer, inner, total), as well as the average of pRNFL thicknesses for 430 participants initially. Later, the study cohort were split into Drop-out and Follow-up groups each 215 individuals. The MMSE scores decreased more rapidly throughout the follow-up period when the total mRNFL thickness at baseline was thinner (*p* = 0.01). When compared to the dropout group, the follow-up group had a moderately thicker retinal layer specifically in the inner mRNFL (*p* = 0.001), total mRNFL (*p* = 0.04), outer GCL outer (*p* = 0.02), and inner INL (*p* = 0.04) at the baseline evaluation.

A stronger decline in the MMSE scores over the follow-up period was correlated with an initial thickness reduction of the total mRNFL at baseline (*p* = 0.01). Moreover, a baseline attenuated outer and inner thicknesses was linked with a decrease in MMSE scores with *p* = 0.04 and *p* = 0.01, respectively. [83] concluded that individuals with a reduced baseline mRNFL were more susceptible to suffer from cognitive impairment compared to their counterparts.

Another longitudinal study by [52] explored the thicknesses of pRNFL (Avg, S, I, T, N), GC-IPL (mean, min, ST, S, SN, IN, I, IT), central macular retinal thickness (MRT), mean macular cube thickness, as well as the macular cube volume for a cohort of 42 AD, 26 aMCI, and 66 HCs. [52] study was keen on obtaining neuropsychological tests for MCI and AD patients once every year for two years through follow ups, hence this study had access to some MCI patients who became AD and were represented by *MCI*_*AD*_. The other group of MCI patients who remained with the same diagnosis, or simply with a stabled cognitive state, were denoted by (*MCI*_*MCI*_). Unlike the 12 *MCI*_*MCI*_ and based on this study, nine *MCI*_*AD*_ had a significant reduction in the T section of pRNFL thickness, the average and minimum GC-IPL thicknesses, and the GC-IPL thickness in the IN, I, and IT sectors. Hence, [52] proved that macular GC-IPL parameters are better in contrast to pRNFL parameters especially in predicting the progression of MCI to AD-type dementia. Conversely, [52] excluded patients with glaucoma, and hence the association between GC-IPL thinning and glaucoma is still unraveled. Simply, GC-IPL thinning might also indicate glaucoma along with AD.

A different longitudinal study by [69] segmented retinal layers by two methods namely N-site Axonal software by Spectralis and a new segmentation technology of the Spectralis. The N-site Axonal software analyzed only the initial cohort of 150 AD, whereas the new segmentation method assessed the retinal layer for follow-ups. They study by [69] initially involved 150 AD patients and 75 age matched HCs; however, only 99 and 51 AD patients were successful in follow-ups<3 years and≥3 years, respectively. The N-site software provided the average pRNFL thickness, the pRNFL thickness ratio between N/T, as well as the thicknesses of papillomacular bundle (PapMac-Bundle) and in quadrants: NS, N, NI, TI, T, and TS. Compared to HCs, the Avg pRNFL, and RNFL in TI, T, TS, PapMac-Bundle, and pRNFL N/T ratio were significantly lower for AD group. On the other hand, the new segmentation method provided the thickness of specific layers ILM, RNFL, GCL, IPL, INL, OPL, ONL, OLM, Photoreceptors, and RPE for various comparisons including: AD versus HCs, AD < 3 years versus AD≥3 years, and HCs versus AD < 3 years versus AD≥3 years. When comparing AD versus HCs, the thicknesses of mRNFL, GCL, IPL, and ONL were notably reduced for AD group conversely to HCs. Surprisingly, both comparisons between AD < 3 years versus AD≥3 as well as the three groups (HCs versus AD < 3 years versus AD≥3 years) were significantly different in mRNFL, GCL, and IPL thicknesses, which indicates that these parameters could prediction the progression of AD.

A longitudinal-based study by [60] focused mainly on IVC FAZ area as well as the annual change of FAZ area between both groups, and these parameters were recorded over the course of 3 consecutive years. Enlarged IVC FAZ was statistically significant (*p* = 0.031) and was associated with *BIOM*_+*ve*_ group. Specifically, the FAZ in *mm*^2^ were 0.272±0.083 and 0.368 ± 0.077 for *BIOM*_-*ve*_ and *BIOM*_+*ve*_, respectively. However, there was no statistically significant change seen at follow-up (*p* > 0.05) for the annual FAZ parameter. A longitudinal research for the course of 2 years in [35] studied OCT parameters (pRNFL, GCC thicknesses) and OCTA parameters (FAZ area, VD of SCP, DCP, CC, and RPC) changes at baseline and after follow-up for aMCI and HCs. The cohort of 18 HCs and 19 aMCI patients were split into 12 stable aMCI (*aMCI*_*Stable*_) patients and 7 aMCI patients converted to (AD-type) dementia (*aMCI*_*Converted*_). Interestingly, the thickness of GCC and pRNFL were significantly reduced for aMCI patients over follow-ups, while no significant changes were noticed for HCs. Additionally, the VD of SCP, DCP, RPC plexus were reduced while FAZ area was increased for aMCI at follow-up in contrast to baseline. Conversely, the VD of CC reduced but did not reach statistical significance between aMCI at baseline and at follow-up. Furthermore, [35] performed a comparison between *aMCI*_*Converted*_ and *aMCI*_*Stable*_ in terms of altered OCT/OCTA parameters. They found out that GCC and pRNFL thicknesses were significantly reduced for both *aMCI*_*Converted*_ and *aMCI*_*Stable*_ sub-categories. In addition, the VD of SCP, DCP, and RPC were reduced, while the FAZ area was increased for *aMCI*_*Stable*_ group at follow-up. On the other hand, only the VD of SCP and RPC where significantly reduced, while the FAZ area was increased for *aMCI*_*Converted*_ group at follow-up. However, there were no significant alterations to the VD of CC in both *aMCI*_*Converted*_ and *aMCI*_*Stable*_ sub-groups, while VD of DCP was also insignificantly changed for only *aMCI*_*Converted*_ patients.

### Diagnosis-related research works

Some research articles attempted to evaluate the diagnostic values for retinal biomarkers in terms of classifying neurodegenerative disorders. Starting with a longitudinal study by [59] which took the course of 25 months. The initial cohort in [59] consisted of 20 MCI and 58 HCs, and the follow-up resulted in 8 HCs converted to MCI, 9 MCI converted to mild-AD, and one MCI patient converted to moderate-AD. [59] combined the stable individuals (from both HCs and MCI groups) to form 60 *Stable* _ *Total* group and combined the converted individuals to form 18 *Converted* _ *Total*. Using baseline story recall as a predictor, the multivariable logistic regression model’s AUC was 0.854; however, when the longitudinal reduction in pRNFL thickness in the inferior quadrant was included, the AUC improved to 0.915. In order to calculate the likelihood of cognitive decline, [59] created a formula based on the pRNFL episodic memory model, and this method produced a conversion score. In comparison to the stable participants, the converted participants’ conversion score was considerably higher (0.59±0.30 versus 0.12±0.19, *p* < 0.001). [59] picked the best conversion score cut-off value of 0.134 which produced a sensitivity and a specificity of 0.944 and 0.767, respectively, in identifying cognitive decline.

Noteworthy, the remaining diagnostic-based studies in this review, [26, 34, 57, 72, 75, 92], involved cross-sectional cohorts only. According to the findings of a study [72], the thickness of the GCL+IPL is the most informative indicator of brain health condition. The study also suggested that a thickness cutoff of 75μm yielded the best balance between sensitivity (85%) and specificity (61%) in a sample comprising 66 individuals with MCI, 27 HCs, and 43 individuals with various types of dementia (including 17 cases of AD, 16 cases of DLB, 6 cases of VCID, and 4 cases of FTD). Individuals with GCL+IPL thickness less than 75μm have lower Montreal Cognitive Assessment scores after Bonferroni correction for multiple comparisons. [72] used logistic regression analysis and found that GCL+IPL thickness had the highest discriminability in distinguishing between healthy controls and individuals with CI, with an AUC of 0.821. The minimum GCL+IPL thickness had the second-highest discriminability, with 0.812 AUC.

In [57], the full retinal thickness (FRT) changes were insignificant between the groups (AD, PD, HCs); however, the issue was mainly caused by mRNFL layer which was then was rolled out due to being a common feature in multiple diseases. Therefore, [57] focused on analyzing texture characteristics of fundus images obtained from OCT scans, and used support vector machines to build a classification model for the purpose of differentiating between the three classes. When building the classification models, removing mRNFL layer achieved better classification results with sensitivity, specificity, and accuracy of 87.0, 100.0, and 82.9, respectively. Participants received the same classification for both eyes with median accuracy percentages of 91.4 (2-fold cross-validation) up to 94.4 (10-fold cross-validation). [57] proved that retinal thickness could be utilized to classify and distinguish between AD, PD, and HCs. The research done by [75] attempted to use logistic regression to distinguish between CH-PAT and CH-NAT groups using the pRNFL thickness in S, I, T, N quadrants as predictors. Their analysis indicated that OD_T and OD_N (OD = right eye) combined predictors resulted in a model with 87% sensitivity and 56% specificity (AUC = 0.83).

Redirecting our attention to research works by [34, 38] that studied both structural and vascular parameters. In [34], the receiver operating characteristics (ROC) curves demonstrated sensitivities (represented as true-positive rate) and specificities (also known as false-positive rate) for various parameters. Also in [34], the area under the ROC (AUC) based on mRNFL+GCL thickness was 0.874, while based on VD parameters was the AUC was 0.696 in terms of differentiating between AD from HCs. Notably, when combining mRNFL+GCL thicknesses as well as the vascular parameters, the ROC curve improved to 0.892 in diagnosing AD patients, and hence better sensitivity. On the contrary, [38] aimed to distinguish between MCI and/or AD with HCs by evaluating the diagnostic performance of retinal VD, measured or compensated cpRNFL, and macular layers (mRNFL, mGCL, and mIPL) parameters. When comparing all groups together, or MCI against HCs, or AD against HCs, or MCI against AD, the retinal VD, and macular layers parameters all failed to draw a line between the groups (*p* > 0.05). Additionally, the compensated cpRNFL thickness also failed to differentiate between MCI and AD groups. However, the compensated cpRNFL thickness parameter surpassed the measured cpRNFL in discriminating between the 3 groups (AUC = 0.74 versus 0.69 with *p* = 0.026), between MCI against HCs (AUC = 0.74 versus 0.68 with *p* = 0.020), and AD against HCs (AUC = 0.79 versus 0.71 with *p* = 0.025). Furthermore, when combining mGCC with either compensated (Model#3) or measured cpRNFL (Model#4), a classification improvement was achieved, compared to the measured cpRNFL, for the 3 groups (AUC = 0.80 versus 0.69 with *p* < 0.001), between MCI against HCs (AUC = 0.79 versus 0.68 with p < 0.001), AD against HCs (AUC = 0.87 versus 0.71 with p < 0.001), and MCI against AD (AUC = 0.72 versus 0.58 with p = 0.003). Finally, mGCC outperformed mGC-IPL to separate MCI and HCs groups (AUC = 0.71 versus 0.66 with p = 0.038); however, mGCC and mGCIP failed to separate AD from HCs due to statistical insignificance.

Interestingly, both [26, 92] research works examined six popular machine learning (ML) techniques, including extreme gradient boosting, light gradient boosting machine, k-nearest neighbor, random forest, gradient boosting, and adaptive boosting, were examined on slightly different cohorts. The accuracy, AUC, f1 score, and recall were the ML assessment metrics that were used. In [26], all six popular ML techniques, were examined on the vascular parameters of 77 AD and 145 HCs cohort. The diagnosis models were developed to include OCTA confounders with statistically significant p-values. The cohort were randomly split into training and testing dataset with a ratio of 7 : 3, where the adaptive boosting algorithm demonstrated the highest diagnostic performance for the testing set with 0.75, 0.73, 0.72, and 0.75 for accuracy, AUC, f1 score, and recall, respectively. On the contrary, in [92], the structural parameters of a cohort composed of 159 AD and 299 HCs were assessed by the same six popular ML techniques used in [26]. Similarly to [26], a random split with 7 : 3 ratio was performed to create training and testing datasets, respectively. The XGBoost algorithm demonstrated the most effective diagnostic performance, with testing scores for the accuracy, AUC, f1 score, and recall of 0.74, 0.69, 0.70, and 0.74, respectively. Except for the Light GBM model, the MRT confounder showed utter superiority in the five ML models. Conversely, the mRNFL thickness was the crucial parameter for the Light GBM model.

## DISCUSSION

The systematic review synthesis concluded potential reasons causing inconsistent results from different research works such as lower cohort number, insignificant parameters only when using specific OCT machines, and biomarkers being ineffective for a cohort with special characteristics (such as EOAD).

The pRNFL-G failed in detecting EOAD [90], indicating that this biomarker is effective only for older AD cohort. Furthermore, the pRNFL-G biomarker may not be suitable for recognizing the progression of neurodegenerative disorders, since it was ineffective in differentiating between moderate AD against mild AD, moderate AD against MCI in [78]. In terms of low cohort number effects, both works by [78, 82] studied the same pRNFL-G thickness parameter and performed a similar comparison between mild AD and HCs; however, there were contradicting results. In fact, there were only 20 mild AD patients in [78] which is considerably lower compared to the 50 mild AD patients in [82]. Hence, this might explain the inconsistent results between both studies [78, 82], where a notable thinning in pRNFL-G thickness was shown in [82], while an insignificant pRNFL-G thickness changes in [78].

Next, we shall investigate effects caused by both lower cohort size conjointly with different OCT machines. Building on the previous analysis, the pRNFL-Avg thickness reduction was considered as an interesting biomarker that was linked with AD patients in contrast to HCs [17, 64, 69, 92]; however, two works by [56, 77] demonstrated negligible pRNFL-Avg variations between AD and HCs groups. Upon further investigation, the OCT machines used in [56, 77] were Cirrus SD-OCT Carl Zeiss (C-SD) and Optos SD-OCT (O-SD), respectively. Interestingly neither C-SD nor O-SD OCT machines were used in the studies by [17, 64, 69, 92] that showed a statistically significant thinning for pRNFL-Avg for AD patients with respect to HCs. Instead, these consensus studies used Cirrus HD-OCT (C-HD), Heidelberg SD-OCT (H-SD), and RTVue-XR Avanti SD-OCT (A-SD) OCT machines. This raises a hypothesis that different OCT machines capture structural variations differently which may have caused this discrepancy rather than questioning the dependability of pRNFL-Avg biomarkers. Therefore, we conclude that pRNFL-Avg thinning is still considered as viable biomarker to discriminate between AD and HCs, except when using C-SD and O-SD OCT machines which should be further investigated in future works. Another potential use for pRNFL-Avg biomarker was found such that a notable thinning was linked with MCI patients compared to HCs [17, 33, 81]; however, insignificant thickness variations of pRNFL-Avg were also reported by [19, 63, 65] between MCI and HCs. When investigating the cause for this inconsistence findings, the pRNFL-Avg thickness variations captured by H-SD [81] and A-SD OCT machines [17, 33] were significantly different between MCI and HCs, with the exception of the findings by [19] which is also captured by A-SD. Noteworthy, only 13 MCI patients were involved in the study by [19], which is significantly smaller than 48 and 54 MCI patients in [17] and [33], respectively. This means that the contradicting results for [19] could be caused by the smaller cohort size rather than the questionability of the pRNFL-Avg biomarker captured by A-SD. After deeper analysis, both contradicting studies by [63, 65] used DRI SS-OCT Triton (T-SS) and C-HD OCT machines. However, the studies by [17, 33, 81], which agreed that pRNFL-Avg significant thinning was indeed associated with MCI patients, did not use neither T-SS nor C-HD OCT devices. Therefore, we infer that pRNFL-Avg thinning can still be regarded as a reliable biomarker to differentiate between MCI and HCs. However, it should be noted that its effectiveness may vary depending on the OCT device used, such as T-SS or C-HD, and the size of the cohort, and most reliable when using A-SD machine. Therefore, more research is warranted to explore these limitations in future studies. Strangely, the *CH*_*NonAgen*_ group had a significantly thinner pRNFL-Avg thickness compared with HCs in [81], and hence the a thinner pRNFL-Avg thickness could be associated with old age (90–100) rather than the cognitive impairments. Therefore, when dealing with nonagenarians, a caution must be considered and the pRNFL-Avg thickness could be misleading.

Now we shift our focus to pRNFL-S biomarker, where a significant thinning was associated with AD patients compared to HCs in [17, 53, 64, 92, 93]; however, only one research findings by [77] did not show any significant changes in pRNFL-S between AD and HCs. After further examination, the C-SD OCT machine used in [77] was not utilized by [17, 53, 64, 92, 93] studies, instead these studies used C-HD, H-SD, and A-SD devices. Therefore, there is a consensus that the pRNFL-S is recognized as a consistent biomarker to detect AD, except when using C-SD OCT machine which requires further investigation in future studies. Regarding the capability of pRNFL-S to detect MCI, the thickness variations of pRNFL-S captured by A-SD in [17, 33] were able to discriminate between MCI and HCs groups; however, some contradicting results were extracted when using H-SD OCT device. Simply, one study by [53] showed a significant thinning of pRNFL-S while another study by [91] demonstrated subtle thickness changes between both MCI and HCs. Additionally, the findings reported by [65, 84] used T-SS and C-HD OCT machines, respectively, which may indicate that there is a limited ability of MCI detection when using these devices. Nevertheless, we conclude a limited MCI detectability for pRNFL-S biomarker which is conditioned by certain OCT machines.

Moving on to another biomarker which is pRNFL-I thickness, where a notable thinning was associated with AD patients in [17, 64, 92] compared to HCs; however, the findings of [53, 77, 93] did report negligible pRNFL-I thickness variations between both groups. Subsequent investigation indicated that the pRNFL-I variations captured by A-SD OCT machine were statistically significant between AD and HCs in [17, 64]. Therefore, we conclude that pRNFL-I biomarker capability in recognizing AD is limited to only A-SD, while unreliable when other OCT machines are used. Both pRNFL-S and pRNFL-I biomarkers were studied together by [61], where both thicknesses were significantly reduced for mmAD comparatively to HCs. Conversely, the finding of [55] which also studied mmAD, were contradicting to [61]. Upon further investigation, the non-significant pRNFL thickness variations could be due to the small cohort of only 20 mmAD in [55], whereas the cohort was 56 mmAD in [61].

We also examined other pRNFL-related biomarkers, where only one study by [69] showed a significant pRNFL-T thinning and another work by [64] yielded a notable pRNFL-N thinning for AD patients compared to HCs. Therefore, the reliability of both pRNFL-T and pRNFL-N in detecting AD is questionable as not enough evidence have been collected.

In terms of general limitations of pRNFL biomarker, it is worth mentioning that thinning of pRNFL, especially the I sector, is a feature associated with both AD and glaucoma [51], and hence this method should be ideally used on patients without glaucoma eye disease to avoid confusion. In other words, if only pRNFL thinning biomarker was used in the analysis, a patient with healthy cognitive abilities yet with glaucoma could be mistakenly diagnosed as AD. Both [51, 52] excluded glaucoma patients from the analysis to avoid facing this issue. A solution came in [33], which included PPG along with aMCI patients. [33] utilized the fact that RPC VD reduction in the whole image was more prominent in PPG group with respect to aMCI. Therefore, the work by [33] used another biomarker, RPC VD reduction, to deal with the shortcomings of pRNFL when dealing with patients with eye pathology of glaucoma.

The subsequent portion of this review will now address other studied biomarkers in the literature including choroidal thickness, FAZ area, and VD loss. In addition, a discussion about some of the reasons that caused difficulties to interpret some of the results. Starting with research works by [20, 55, 71, 88] that proved the effectiveness of choroidal thickness as a retinal biomarker to distinguish between AD and HCs. However, the use of choroidal thickness evaluation as a potential diagnostic tool in AD should be restricted to non-AMD patients because choroidal thinning is a trait common to both AMD and AD in the elderly [96]. Now, redirecting our attention to another biomarker, FAZ enlargement at IVC was linked with *BIOM*_+*ve*_ group, MCI and AD, and ATD groups in [20, 21, 60], respectively. However, an exception was documented in [16], where minimal changes in IVC FAZ were observed in the combined aMCI/eAD group. We hypnotize that this discrepant results could be caused by the low cohort (13 aMCI and 3 eAD) used by [16], in comparison to the cohort (21 MCI and 18 AD) used by [21]. FAZ enlargement at IVC was linked with *BIOM*_+*ve*_ group, MCI and AD, and ATD groups in [20, 21, 60], respectively. However, an exception was documented in [16], where minimal changes in IVC FAZ were observed in the combined aMCI/eAD group. We hypnotize that this discrepant results could be caused by the low cohort (13 aMCI and 3 eAD) used by [16], in comparison to the cohort (21 MCI and 18 AD) used by [21]. Shifting our focus to a different biomarker, the reduction of DCP-VD was also thought to be a relevant indicator associated with AD, MCI, and *APOE* ɛ4 + groups in [21, 22], and [24] respectively. However, other contradicting findings by only one research work [25] was also documented. We hypothesized this inconsistency is caused by the very low cohort number of 7 AD patients. In addition to the previously mentioned causes for conflicting findings, interpreting some of the results was challenging due to the dissimilar layer segmentation definitions, as shown in Table 1. Although the promising results by [20] indicated a notable microvascular loss for AD patients compared to HCs in some sectors of SCP and DCP; however, the layer definition used by [20] were very distinct to compared to the classical layer definitions, as shown in Table 1. In addition, only the work by [20] used this special layer definition, and no other research work to our knowledge used the same layer definitions and attempted to find association between VD parameters and AD patients. Hence, although the promising results by [20], there are not enough evidence in the literature to support this biomarker.

We will now turn our attention to the diagnostic potential of retinal biomarkers in the context of identifying different cognitive decline disorders. The longitudinal study by [59] initially used the baseline story recall as a predictor, and the multivariable logistic regression model resulted in 0.854 AUC. However, when the longitudinal reduction of pRNFL thickness in the inferior quadrant was included as a predictor, the AUC improved to 0.915. These results imply the importance of longitudinal data, specifically monitoring pRNFL changes.

The next diagnostic works by [26, 34, 57, 72, 75, 92] involved cross-sectional cohorts. Research studied by [34, 57, 72, 92] studied different structural retinal thicknesses and their diagnostic impact. For instance, the AUC based on mRNFL+GCL thickness was 0.874 in [34] distinguishing between AD and HCs. Moreover, the full structural retinal thickness, excluding mRNFL, led to sensitivity, specificity, and accuracy of 87.0, 100.0, and 82.9, respectively, when comparing AD, PD, and HCs in [57]. Additionally, the study by [72] showed that GCL+IPL thickness had the highest discriminability power discriminating between cognitively impaired and HCs with an AUC of 0.821, and a thickness cut-off of 75μm for GCL+IPL yielded the best balance between sensitivity (85%) and specificity (61%). Also, the structural parameters with statistically significant p-values were fed to XGBoost algorithm in [92] which resulted in accuracy, AUC, f1 score, and recall of 0.74, 0.69, 0.70, and 0.74, respectively. Although these results indicate the potential of structural thicknesses in identifying cognitive decline; however, the comparison between such studies is rather unfair due to the different cohorts and studied layers, as well as the various used techniques. Another logistic regression analysis by [75] indicated that OD_T and OD_N (OD = right eye) combined pRNFL thicknesses predictors resulted in a model with 87% sensitivity and 56% specificity (AUC = 0.83). This confirms the great potential of pRNFL biomarker in the diagnosis between CH-PAT and CH-NAT groups; however, this result must be further explored with different cohorts and OCT machines.

In terms of the diagnostic-based studies that explored OCTA parameters, the VD parameters in [34] led to an AUC of 0.696 differentiating between AD from HCs. Moreover, the diagnosis models in [26] were developed to include OCTA confounders with statistically significant p-values, where the adaptive boosting algorithm demonstrated the highest diagnostic performance for the testing set with 0.75, 0.73, 0.72, and 0.75 for accuracy, AUC, f1 score, and recall, respectively. These findings indicate the viability of OCTA parameter in detecting cognitive degeneration; however, future research work must be investigated as not enough evidence in the literature.

Lastly, only one study by [34], as per our knowledge, attempted to explore the diagnostic potential by studying mRNFL+GCL thicknesses as well as the vascular parameters conjointly, which led to an improved AUC of 0.892 in diagnosing AD patients. Therefore, the incorporation of diverse parameters has the potential to enhance the diagnostic efficacy since the merged parameters’ result is better than the AUCs of 0.696 and 0.874 for VD and mRNFL+GCL parameters, respectively.

### Concluding remarks

The inclusion and exclusion criteria for the review, illustrated in Fig. 1, led to the incorporation of 64 original papers included in this investigation. The scope of this study involves non-invasive imaging techniques OCT & OCTA and their potential in diagnosing dementia disorders. Furthermore, the most prevalent dementia diseases, including AD, VaD, DLB, FTD, MixDem, and a few PD-based papers with AD/MCI, were simply described in this paper.

The results of the current work indicate that the pRNFL-S is a trustworthy biomarker to detect most AD cases; however, its effectiveness may be impacted when using a C-SD OCT machine, and hence, additional research is needed in future studies. In addition, the pRNFL-S biomarker showed no discriminative power between mild AD and HCs in [87, 92]. Similarly, the dependability of pRNFL-S as a biomarker for detecting MCI is also restricted by specific OCT machines, such that the optimal effectiveness is achieved when using A-SD OCT machines. Furthermore, recent studies confirmed the capability of pRNFL-I biomarker in recognizing AD is limited to only A-SD [17, 64], while deemed unreliable when other OCT machines are used.

Another reliable biomarker, the pRNFL-G, effectively differentiates between FTD from mild AD and HCs in [78], moderate AD from HCs in [78], MCI from HCs in [53, 78], as well as detecting pathological Aβ_42_/tau in cognitively healthy individuals [75]. Yet, the pitfall of the pRNFL-G biomarker is the contradictory results in findings by [78] and [82]. Simply, a significant thinning of pRNFL-G was linked with mild AD in [82], while negligible pRNFL-G thickness changes were documented in [78] between mild AD and HCs. Another limitation of pRNFL-G is its inability to recognize the progression of neurodegenerative disorders [78], and hence was also not capable to discriminate between multiple groups with varied degrees of cognitive impairment [50, 66].

In addition to the previously discussed biomarkers, emerging evidence suggests that the pRNFL-Avg thinning is considered as viable biomarker to discriminate between AD and HCs, except when using C-SD and O-SD OCT machines which should be further investigated in future works. Additionally, the pRNFL-Avg was capable of discriminating between severe and mild AD [92], and hence, assessing the worsening stage of AD. Moreover, pRNFL-Avg thinning could be regarded as a dependable biomarker to differentiate between MCI and HCs; however, its effectiveness is questionable when using DRI SS-OCT Triton-Topcon (T-SS) or C-HD OCT machines and when the size of the cohort is relatively low.

It is also worth noting that recent studies by [20, 55, 71, 88] proved the robustness of choroidal thickness as a retinal biomarker to differentiate between AD and HCs groups. On the contrary, the utilization of choroidal thickness should be limited to non-AMD patients, because choroidal thinning is a trait shared by both AMD and AD in the elderly [96].

Pivoting our focus to another category of biomarkers, vasculature-based changes that are linked with cognitive impairment include FAZ enlargement, as well as alterations around FAZ, VD reduction in ICP and DCP (VPD and VLD) for cognitively impaired individuals in contrast to HCs. However, there were no sufficient evidence to support this correlation, as only a few works reported significant vasculature variations while other works were contradicting. Additionally, the process of comparing the vascular-based parameters was challenging due to the various layers’ definitions adopted by researchers, where a summary was previously shown in Table 1. With regards to the enlargement of FAZ area, a few things must be considered. Firstly, the retinal layers involved prior retrieving En-Face OCTA image projection, for instance, the FAZ of IVC will be much different than SCP. Secondly, the FAZ parameters extracted by various OCT machines could be different due to the hardware operation differences of these machines or related to the segmentation methods used to extract FAZ area. Thirdly, FAZ parameters are affected by other factors like myopic eyes, and these parameters could be adjusted with axial length information [97]. Bearing in mind the previous limitations, the FAZ area changes has limited capabilities to detect cognitive impairment.

A general problem was identified by the review which is the unavailability of certain parameters. Unlike the pRNFL thickness, the process of determining the appropriate retinal layers to identify neurodegenerative disorders is quite challenging due to the discrepancy between the findings. Simply, different works studied some parameters which are not found in other research articles. For instance, GC-IPL thickness was studied in [98] but not in all research articles in the literature.

The diagnostic capabilities of various retinal biomarkers have been explored in this review. Most of these biomarkers are based on structural thicknesses, while other biomarkers are based on vascular changes. On the other hand, we hypothesize that combining multiple OCT/OCTA biomarkers will enhance the overall diagnostic accuracy for the task of recognizing distinct neurodegenerative disorders. Our assumptions were supported by [34], where the incorporation of both structural thicknesses and vascular parameters yielded in a better AUC results, and consequently, this would potentially be the future direction of this field of study.

The potential limitation of this review could be the fact of not involving pre-clinical AD and focusing on other works related to dementia. Based on the discussed methods of extracting retinal structural and/or vascular parameters, there are a few points to be considered. In short, the segmentation of both retinal vasculature and layers is a crucial step before extracting retinal parameters. Errors in segmentation may lead to incorrect extracted parameters and hence the retinal analysis may fail to realize significant outcomes or the outcome could be misleading. Researchers tend to perform manual corrections to the segmented images; however, this process is not only time-consuming but also requires great expertise in the medical field which potentially not feasible. Therefore, next future work should focus on developing and improving segmentation approaches to efficiently/accurately automate the procedure of retinal layers extraction. Noteworthy, improving the segmentation efficacy would open the door to investigate more biomarkers that initially thought to be irrelevant to various disorders.

## Supplementary Material

Supplementary Table 1

Supplementary Table 2

Supplementary Table 3

## Data Availability

This article did not involve the generation or analysis of datasets; hence, data sharing is not applicable.
