# Supplementary material for: A Systematic Review on Retinal Biomarkers to Diagnose Dementia from OCT/OCTA Images
Source: J Alzheimers Dis Rep. 2023 Nov 1;7(1):1201–35. doi: 10.3233/ADR-230042 (PMC10657718; doi:10.3233/ADR-230042)
Supplement: Supplementary Table 1 [file adr-7-adr230042-s001.docx]

**Supplementary Table 1. Cross-Sectional Macula-Related Parameters**

|  | FRT | ILM | mRNFL | GCL | IPL | mRNFL + GCL | GC-IPL | GCC | INL | OPL | ONL | ELM/ OLM | PhotoR | IS-OS/EZ | OSL | IZ | RPE | OPR | Macula volume | GCL Volume | IPL Volume | INL Volume | Outer Retina |
| --- | --- | --- | --- | --- | --- | --- | --- | --- | --- | --- | --- | --- | --- | --- | --- | --- | --- | --- | --- | --- | --- | --- | --- |
| **AD** |  |  |  |  |  |  |  |  |  |  |  |  |  |  |  |  |  |  |  |  |  |  |  |
| **AD vs. HCs:** |  |  |  |  |  |  |  |  |  |  |  |  |  |  |  |  |  |  |  |  |  |  |  |
| [1] AD: 159 vs. HCs: 299 | $-$ | $-$ | $-$ | $\downarrow$ | $\downarrow$ | $-$ | $-$ | $-$ | $-^{NS}$ | $-^{NS}$ | $-^{NS}$ | $-$ | $-$ | $-^{NS}$ | $-^{NS}$ | $-$ | $-^{NS}$ | $-^{NS}$ | $-$ | $-$ | $-$ | $-$ | $-$ |
| [2] AD: 7, HCs: 8 | $-$ | $-$ | $-$ | $-$ | $-$ | $-$ | $\downarrow$ | $-$ | $-$ | $-$ | $-$ | $-$ | $-$ | $-$ | $-$ | $-$ | $-$ | $-$ | $-$ | $-$ | $-$ | $-$ | $-$ |
| [3] AD: 21 vs. HCs: 25 | $-$ | $-$ | $-$ | $-$ | $-$ | $-$ | $-$ | $-$ | $-$ | $-$ | $-$ | $-$ | $-$ | $-$ | $-$ | $-$ | $-$ | $-$ | $-$ | $-$ | $-$ | $-$ | $-$ |
| [4] AD: 20 vs. HCs: 28 | $-$ | $-$ | $-$ | $-$ | $-$ | $-$ | $-$ | $-$ | $-$ | $-$ | $-$ | $-$ | $-$ | $-$ | $-$ | $-$ | $-$ | $-$ | $-$ | $-$ | $-$ | $-$ | $-$ |
| [5] AD: 42 vs. HCs: 45 | $-$ | $-$ | $-$ | $-$ | $-$ | $-$ | $-$ | $\downarrow$ | $-$ | $-$ | $-$ | $-$ | $-$ | $-$ | $-$ | $-$ | $-$ | $-$ | $-$ | $-$ | $-$ | $-$ | $-$ |
| [6] tAD: 23 vs. HCs: 70 | $-^{NS}$ | $-$ | $-$ | $-$ | $-$ | $-$ | $-$ | $-$ | $-$ | $-$ | $-$ | $-$ | $-$ | $-$ | $-$ | $-$ | $-$ | $-$ | $-$ | $-$ | $-$ | $-$ | $-$ |
| [7] AD: 150, HCs: 75 | $-^{NS}$ | $-^{NS}$ | $\downarrow$ | $\downarrow$ | $\downarrow$ | $-$ | $-$ | $-$ | $-^{NS}$ | $-^{NS}$ | $\downarrow$ | $-^{NS}$ | $-$ | $-$ | $-$ | $-$ | $-^{NS}$ | $-$ | $-$ | $-$ | $-$ | $-$ | $-$ |
| [8] AD: 43 vs. HCs: 57 | $-$ | $-$ | $-$ | $-$ | $-$ | $-$ | $-$ | $-$ | $-$ | $-$ | $-$ | $-$ | $-$ | $-$ | $-$ | $-$ | $-$ | $-$ | $-$ | $\downarrow$ | $\downarrow$ | $-^{NS}$ | $-$ |
| **Mild AD vs. HCs:** |  |  |  |  |  |  |  |  |  |  |  |  |  |  |  |  |  |  |  |  |  |  |  |
| [9] mild AD: 19 vs. HCs: 24 (Mean thickness) $\xi$ | $-^{NS}$ | $-$ | $-^{NS}$ | $-^{NS}$ | $-^{NS}$ | $-$ | $-$ | $-$ | $-^{NS}$ | $-^{NS}$ | $-^{NS}$ | $-$ | $-$ | $-^{NS}$ | $\downarrow$ | $-$ | $-^{NS}$ | $-^{NS}$ | $-$ | $-$ | $-$ | $-$ | $-$ |
| [9] mild AD: 19 vs.  HCs: 24 (point by point)$\xi$ | $\downarrow$ | $-$ | $\downarrow$ | $\downarrow$ | $\downarrow$ | $-$ | $-$ | $-$ | $\downarrow$ | $-^{NS}$ | $-^{NS}$ | $-$ | $-$ | $-^{NS}$ | $\downarrow$ | $-$ | $-^{NS}$ | $-^{NS}$ | $-$ | $-$ | $-$ | $-$ | $-$ |
| [10] 19 mild AD and 24 HCs. | $-^{NS}$ | $-$ | $\downarrow$ | $-^{NS}$ | $-^{NS}$ | $-$ | $-$ | $-$ | $\downarrow$ | $-^{NS}$ | $-^{NS}$ | $-$ | $-$ | $-^{NS}$ | $\downarrow$ | $-$ | $-^{NS}$ | $-^{NS}$ | $-$ | $-$ | $-$ | $-$ | $-$ |
| [1] Mild AD: 51 vs. HCs: 299 | $-$ | $-$ | $-$ | $\downarrow$ | $\downarrow$ | $-$ | $-$ | $-$ | $-^{NS}$ | $-^{NS}$ | $-^{NS}$ | $-$ | $-$ | $-^{NS}$ | $-^{NS}$ | $-$ | $-^{NS}$ | $-^{NS}$ | $-$ | $-$ | $-$ | $-$ | $-$ |
| [11] Mild AD: 20 vs. HCs: 49 | $-$ | $-$ | $-$ | $-$ | $-$ | $-$ | $-^{NS}$ | $-$ | $-$ | $-$ | $-$ | $-$ | $-$ | $-$ | $-$ | $-$ | $-$ | $-$ | $-$ | $-$ | $-$ | $-$ | $-$ |
| [65] Mild AD: 37, HCs: 29 | $-$ | $-$ | $-$ | $-$ | $-$ | $-$ | $-$ | $-$ | $-$ | $-$ | $-$ | $-$ | $-$ | $-$ | $-$ | $-$ | $-$ | $-$ | $-$ | $-$ | $-$ | $-$ | $-$ |
| **Moderate/Severe AD** |  |  |  |  |  |  |  |  |  |  |  |  |  |  |  |  |  |  |  |  |  |  |  |
| [1] Moderate AD: 67, vs. HCs: 299 | $-$ | $-$ | $-$ | $\downarrow$ | $\downarrow$ | $-$ | $-$ | $-$ | $-^{NS}$ | $-^{NS}$ | $-^{NS}$ | $-$ | $-$ | $-^{NS}$ | $-^{NS}$ | $-$ | $-^{NS}$ | $-^{NS}$ | $-$ | $-$ | $-$ | $-$ | $-$ |
| [11] Mod AD: 17 vs. HCs: 49 | $-$ | $-$ | $-$ | $-$ | $-$ | $-$ | $\downarrow$ | $-$ | $-$ | $-$ | $-$ | $-$ | $-$ | $-$ | $-$ | $-$ | $-$ | $-$ | $-$ | $-$ | $-$ | $-$ | $-$ |
| [1] Severe AD: 41 vs. HCs: 299 | $-$ | $-$ | $-$ | $\downarrow$ | $\downarrow$ | $-$ | $-$ | $-$ | $-^{NS}$ | $-^{NS}$ | $-^{NS}$ | $-$ | $-$ | $-^{NS}$ | $-^{NS}$ | $-$ | $-^{NS}$ | $-^{NS}$ | $-$ | $-$ | $-$ | $-$ | $-$ |
| **MCI** |  |  |  |  |  |  |  |  |  |  |  |  |  |  |  |  |  |  |  |  |  |  |  |
| **MCI vs. HCs:** |  |  |  |  |  |  |  |  |  |  |  |  |  |  |  |  |  |  |  |  |  |  |  |
| [5] MCI: 48 vs. HCs: 45 | $-$ | $-$ | $-$ | $-$ | $-$ | $-$ | $-$ | $\downarrow$ | $-$ | $-$ | $-$ | $-$ | $-$ | $-$ | $-$ | $-$ | $-$ | $-$ | $-$ | $-$ | $-$ | $-$ | $-$ |
| [12] MCI: 24 vs. HCs: 31 | $-^{NS}$ | $-$ | $-^{NS}$ | $-^{NS}$ | $-^{NS}$ | $-$ | $-$ | $-$ | $-^{NS}$ | $-^{NS}$ | $-^{NS}$ | $-$ | $-$ | $-$ | $-$ | $-$ | $-$ | $-^{NS}$ | $-$ | $-$ | $-$ | $-$ | $-$ |
| [13] aMCI: 17 vs. HCs: 17 | $-$ | $-$ | $-$ | $-$ | $-$ | $-$ | $-^{NS}$ | $-$ | $-$ | $-$ | $-$ | $-$ | $-$ | $-$ | $-$ | $-$ | $-$ | $-$ | $-^{NS}$ | $-$ | $-$ | $-$ | $-$ |
| [14] non-amnestic MCI: 17 vs. HCs: 56 | $-$ | $-$ | $-$ | $-$ | $-$ | $-$ | $-^{NS}$ | $-$ | $-$ | $-$ | $-$ | $-$ | $-$ | $-$ | $-$ | $-$ | $-$ | $-$ | $-$ | $-$ | $-$ | $-$ | $-^{NS}$ |
| [14] amnestic MCI: 59 vs. HCs: 56 | $-$ | $-$ | $-$ | $-$ | $-$ | $-$ | $-^{NS}$ | $-$ | $-$ | $-$ | $-$ | $-$ | $-$ | $-$ | $-$ | $-$ | $-$ | $-$ | $-$ | $-$ | $-$ | $-$ | $-^{NS}$ |
| [11] MCI: 29 vs. HCs: 49 | $-$ | $-$ | $-$ | $-$ | $-$ | $-$ | $-^{NS}$ | $-$ | $-$ | $-$ | $-$ | $-$ | $-$ | $-$ | $-$ | $-$ | $-$ | $-$ | $-$ | $-$ | $-$ | $-$ | $-$ |
| [15] CKD_Low: 13 vs. CKD_High: 99 | $-$ | $-$ | $-$ | $-^{NS}$ | $-$ | $-$ | $-$ | $-$ | $-$ | $-$ | $-$ | $-$ | $-$ | $-$ | $-$ | $-$ | $-$ | $-$ | $-$ | $-$ | $-$ | $-$ | $-$ |
| [16] aMCI: 54 vs. HCs: 54 | $-$ | $-$ | $-$ | $-$ | $-$ | $-$ | $-$ | $\downarrow$ | $-$ | $-$ | $-$ | $-$ | $-$ | $-$ | $-$ | $-$ | $-$ | $-$ | $-$ | $-$ | $-$ | $-$ | $-$ |
| [17] aMCI: 23 vs. HCs: 24 | $-^{NS}$ | $-$ | $-$ | $-$ | $-$ | $-$ | $-$ | $-$ | $-$ | $-$ | $-$ | $-$ | $-$ | $-$ | $-$ | $-$ | $-$ | $-$ | $-$ | $-$ | $-$ | $-$ | $-$ |
| [8] MCI: 37 vs. HCs: 57 | $-$ | $-$ | $-$ | $-$ | $-$ | $-$ | $-$ | $-$ | $-$ | $-$ | $-$ | $-$ | $-$ | $-$ | $-$ | $-$ | $-$ | $-$ | $-$ | $-^{NS}$ | $-^{NS}$ | $-^{NS}$ | $-$ |
| **Multi-Comparisons:** |  |  |  |  |  |  |  |  |  |  |  |  |  |  |  |  |  |  |  |  |  |  |  |
| [18] Mild AD: 22 vs. Mod AD: 16 vs. HCs: 62 | $-^{NS}$ | $-$ | $-$ | $-$ | $-$ | $-$ | $-$ | $-$ | $-$ | $-$ | $-$ | $-$ | $-$ | $-$ | $-$ | $-$ | $-$ | $-$ | $-$ | $-$ | $-$ | $-$ | $-$ |
| [19] MCI: 66 vs. Dementia: 43 vs. HCs: 27 | $-$ | $-$ | $-$ | $-$ | $-$ | $-$ | $\downarrow$ | $-$ | $-$ | $-$ | $-$ | $-$ | $-$ | $-$ | $-$ | $-$ | $-$ | $-$ | $-^{NS}$ | $-$ | $-$ | $-$ | $-$ |
| [19] MCI: 66 vs. AD: 17 vs. DLBs: 16 vs. VCID: 6 vs. FTD: 4 vs. HCs: 27 | $-$ | $-$ | $-$ | $-$ | $-$ | $-$ | $\downarrow$ | $-$ | $-$ | $-$ | $-$ | $-$ | $-$ | $-$ | $-$ | $-$ | $-$ | $-$ | $\downarrow$ | $-$ | $-$ | $-$ | $-$ |
| [14] amnestic MCI: 59 vs. non-amnestic MCI: 17 vs. HCs: 56 | $-$ | $-$ | $-$ | $-$ | $-$ | $-$ | $-^{NS}$ | $-$ | $-$ | $-$ | $-$ | $-$ | $-$ | $-$ | $-$ | $-$ | $-$ | $-$ | $-$ | $-$ | $-$ | $-$ | $-$ |
| [20] MCI: 15, mmAD: 15, HCs: 18 | $-$ | $-$ | $-^{NS}$ | $-$ | $-$ | $-$ | $-^{NS}$ | $-$ | $-$ | $-$ | $-$ | $-$ | $-$ | $-$ | $-$ | $-$ | $-$ | $-$ | $-$ | $-$ | $-$ | $-$ | $-$ |
| [21] aMCI: 192, AD: 324, HCs: 414 | $-^{NS}$ | $-$ | $-^{NS}$ | $-^{NS}$ | $-$ | $-$ | $-$ | $-$ | $-$ | $-$ | $-$ | $-$ | $-$ | $-$ | $-$ | $-$ | $-$ | $-$ | $-^{NS}$ | $-$ | $-$ | $-$ | $-$ |

|  | FRT | ILM | mRNFL | GCL | IPL | mRNFL + GCL | GC-IPL | GCC | INL | OPL | ONL | ELM/ OLM | PhotoR | IS-OS/EZ | OSL | IZ | RPE | OPR | Macula volume | GCL Volume | IPL Volume | INL Volume | Outer Retina |
| --- | --- | --- | --- | --- | --- | --- | --- | --- | --- | --- | --- | --- | --- | --- | --- | --- | --- | --- | --- | --- | --- | --- | --- |
| **Special Comparison** |  |  |  |  |  |  |  |  |  |  |  |  |  |  |  |  |  |  |  |  |  |  |  |
| [22] EOAD: 15, HCs: 15 | $-^{NS}$ | $-$ | $-$ | $-$ | $-$ | $-$ | $-$ | $-$ | $-$ | $-$ | $-$ | $-$ | $-$ | $-$ | $-$ | $-$ | $-$ | $-$ | $-$ | $-$ | $-$ | $-$ | $-$ |
| [1] Severe AD: 41 vs. Mild AD: 51 | $-$ | $-$ | $-$ | $-^{NS}$ | $-^{NS}$ | $-$ | $-$ | $-$ | $-^{NS}$ | $-^{NS}$ | $-^{NS}$ | $-$ | $-$ | $-^{NS}$ | $-^{NS}$ | $-$ | $-^{NS}$ | $-^{NS}$ | $-$ | $-$ | $-$ | $-$ | $-$ |
| [1] Severe AD: 41 vs. Moderate AD: 67 | $-$ | $-$ | $-$ | $-^{NS}$ | $-^{NS}$ | $-$ | $-$ | $-$ | $-^{NS}$ | $-^{NS}$ | $-^{NS}$ | $-$ | $-$ | $-^{NS}$ | $-^{NS}$ | $-$ | $-^{NS}$ | $-^{NS}$ | $-$ | $-$ | $-$ | $-$ | $-$ |
| [14] amnestic MCI: 59 vs. non-amnestic MCI: 17 | $-$ | $-$ | $-$ | $-$ | $-$ | $-$ | $-^{NS}$ | $-$ | $-$ | $-$ | $-$ | $-$ | $-$ | $-$ | $-$ | $-$ | $-$ | $-$ | $-$ | $-$ | $-$ | $-$ | $-^{NS}$ |
| [11] FTD: 17 vs. HCs: 49 | $-$ | $-$ | $-$ | $-$ | $-$ | $-$ | $\downarrow$ | $-$ | $-$ | $-$ | $-$ | $-$ | $-$ | $-$ | $-$ | $-$ | $-$ | $-$ | $-$ | $-$ | $-$ | $-$ | $-$ |
| [11] FTD: 17 vs. Mild AD: 20 | $-$ | $-$ | $-$ | $-$ | $-$ | $-$ | $\downarrow$ | $-$ | $-$ | $-$ | $-$ | $-$ | $-$ | $-$ | $-$ | $-$ | $-$ | $-$ | $-$ | $-$ | $-$ | $-$ | $-$ |
| [11] Mod AD: 17 vs. MCI: 29 | $-$ | $-$ | $-$ | $-$ | $-$ | $-$ | $\downarrow$ | $-$ | $-$ | $-$ | $-$ | $-$ | $-$ | $-$ | $-$ | $-$ | $-$ | $-$ | $-$ | $-$ | $-$ | $-$ | $-$ |
| [11] Mod AD: 17 vs. Mild AD: 20 | $-$ | $-$ | $-$ | $-$ | $-$ | $-$ | $\downarrow$ | $-$ | $-$ | $-$ | $-$ | $-$ | $-$ | $-$ | $-$ | $-$ | $-$ | $-$ | $-$ | $-$ | $-$ | $-$ | $-$ |
| [23] FTD: 16 vs. HCs: 30 | $-^{NS}$ | $-$ | $-^{NS}$ | $-^{NS}$ | $-^{NS}$ | $-$ | $-$ | $-$ | $-^{NS}$ | $\downarrow$ | $\downarrow$ | $-$ | $-^{NS}$ | $\downarrow$ | $-$ | $-^{NS}$ | $-^{NS}$ | $-$ | $-$ | $-$ | $-$ | $-$ | $\downarrow$ |
| [23] FTD: 16 vs. HCs: 30 $*^{AdjX}$ | $-^{NS}$ | $-$ | $-^{NS}$ | $-^{NS}$ | $-^{NS}$ | $-$ | $-$ | $-$ | $-^{NS}$ | $-^{NS}$ | $\downarrow$ | $-$ | $-^{NS}$ | $\downarrow$ | $-$ | $-^{NS}$ | $-^{NS}$ | $-$ | $-$ | $-$ | $-$ | $-$ | $\downarrow$ |
| [24] FTD: 27 vs. HCs: 44 | $-^{NS}$ | $-$ | $-^{NS}$ | $-^{NS}$ | $-^{NS}$ | $-$ | $-$ | $-$ | $-^{NS}$ | $\downarrow$ | $\downarrow$ | $-$ | $-$ | $\downarrow$ | $-$ | $\downarrow$ | $-^{NS}$ | $-$ | $-$ | $-$ | $-$ | $-$ | $-$ |
| [24] FTD: 27 vs. HCs: 44 $*^{AdjX}$ | $-^{NS}$ | $-$ | $-^{NS}$ | $-^{NS}$ | $-^{NS}$ | $-$ | $-$ | $-$ | $-^{NS}$ | $-^{NS}$ | $\downarrow$ | $-$ | $-$ | $\downarrow$ | $-$ | $-^{NS}$ | $-^{NS}$ | $-$ | $-$ | $-$ | $-$ | $-$ | $-$ |
| [25] AD Dementia:14 vs. PD: 19 vs. HCs: 31 | $-^{NS}$ | $-$ | $-$ | $-$ | $-$ | $-$ | $-$ | $-$ | $-$ | $-$ | $-$ | $-$ | $-$ | $-$ | $-$ | $-$ | $-$ | $-$ | $-^{NS}$ | $-$ | $-$ | $-$ | $-$ |
| **S****upported by CSF and/or Brain MRI** | | | | |  |  |  |  |  |  |  |  |  |  |  |  |  |  |  |  |  |  |  |
| [26] ${BIOM}_{+ve}$: 9 vs. ${BIOM}_{-ve}$: 11 | $-$ | $-$ | $-^{NS}$ | $-^{NS}$ | $-$ | $-$ | $-$ | $-$ | $-$ | $-$ | $-$ | $-$ | $-$ | $-$ | $-$ | $-$ | $-$ | $-$ | $-$ | $-$ | $-$ | $-$ | $-$ |
| [27] CH-PAT: 27 vs. CH-NAT: 16 | $-^{NS}$ | $-$ | $-$ | $-$ | $-$ | $-$ | $-^{NS}$ | $-$ | $-$ | $-$ | $-$ | $-$ | $-$ | $-$ | $-$ | $-$ | $-$ | $-$ | $-$ | $-$ | $-$ | $-$ | $-$ |
| [12] APOE-ε4+:10 vs. APOE-ε4-: 18 | $-^{NS}$ | $-$ | $-^{NS}$ | $-^{NS}$ | $-^{NS}$ | $-$ | $-$ | $-$ | $-^{NS}$ | $-^{NS}$ | $-^{NS}$ | $-$ | $-$ | $-$ | $-$ | $-$ | $-^{NS}$ | $-$ | $-$ | $-$ | $-$ | $-$ | $-$ |
| [24] Probable tauopathy: 19 vs. HCs: 44 | $-^{NS}$ | $-$ | $-$ | $-$ | $-$ | $-$ | $-$ | $-$ | $-$ | $-^{NS}$ | $\downarrow$ | $-$ | $-$ | $\downarrow$ | $-$ | $-$ | $-$ | $-$ | $-$ | $-$ | $-$ | $-$ | $-$ |
| [24] TDP-43: 2 vs. HCs: 44 | $-^{NS}$ | $-$ | $-$ | $-$ | $-$ | $-$ | $-$ | $-$ | $-$ | $-^{NS}$ | $-^{NS}$ | $-$ | $-$ | $-^{NS}$ | $-$ | $-$ | $-$ | $-$ | $-$ | $-$ | $-$ | $-$ | $-$ |
| [24] Unknown pathology: 6 vs. HCs: 44 | $-^{NS}$ | $-$ | $-$ | $-$ | $-$ | $-$ | $-$ | $-$ | $-$ | $-^{NS}$ | $\downarrow$ | $-$ | $-$ | $-^{NS}$ | $-$ | $-$ | $-$ | $-$ | $-$ | $-$ | $-$ | $-$ | $-$ |
| [23] tauopathy: 9 vs. HCs: 30 | $\downarrow$ | $-$ | $-^{NS}$ | $-^{NS}$ | $-^{NS}$ | $-$ | $-$ | $-$ | $-^{NS}$ | $-^{NS}$ | $\downarrow$ | $-$ | $-^{NS}$ | $\downarrow$ | $-$ | $-^{NS}$ | $-^{NS}$ | $-$ | $-$ | $-$ | $-$ | $-$ | $\downarrow$ |
| [23] tauopathy: 9 vs. HCs: 30 $*^{AdjX}$ | $-^{NS}$ | $-$ | $-^{NS}$ | $-^{NS}$ | $-^{NS}$ | $-$ | $-$ | $-$ | $-^{NS}$ | $-^{NS}$ | $-^{NS}$ | $-$ | $-^{NS}$ | $\downarrow$ | $-$ | $-^{NS}$ | $-^{NS}$ | $-$ | $-$ | $-$ | $-$ | $-$ | $\downarrow$ |

Noteworthy, all tables illustrate significant changes by $\uparrow$ or $\downarrow$ arrows, insignificant changes by $-^{NS}$, and $-$ for any missing parameters.

See Table 2 for definitions of abbreviations.

**REFERENCES**

[1] Wang X, Jiao B, Liu H, Wang Y, Hao X, Zhu Y, Xu B, Xu H, Zhang S, Jia X (2022) Machine learning based on Optical Coherence Tomography images as a diagnostic tool for Alzheimer's disease. *CNS Neurosci Therap* **28**, 2206-2217.

[2] Sadda SR, Borrelli E, Fan W, Ebraheem A, Marion KM, Harrington M, Kwon S (2019) A pilot study of fluorescence lifetime imaging ophthalmoscopy in preclinical Alzheimer’s disease. *Eye* **33**, 1271-1279.

[3] Poroy C, Yücel AÂ (2018) Optical coherence tomography: Is really a new biomarker for alzheimer's disease? *Ann Indian Acad Neurol* **21**, 119.

[4] Garcia-Martin ES, Rojas B, Ramirez AI, de Hoz R, Salazar JJ, Yubero R, Gil P, Triviño A, Ramirez JM (2014) Macular thickness as a potential biomarker of mild Alzheimer's disease. *Ophthalmology* **121**, 1149-1151. e1143.

[5] Tao R, Lu Z, Ding D, Fu S, Hong Z, Liang X, Zheng L, Xiao Y, Zhao Q (2019) Perifovea retinal thickness as an ophthalmic biomarker for mild cognitive impairment and early Alzheimer's disease. *Alzheimers Dement (Amst)* **11**, 405-414.

[6] den Haan J, Csinscik L, Parker T, Paterson RW, Slattery CF, Foulkes A, Bouwman FH, Verbraak FD, Scheltens P, Peto T (2019) Retinal thickness as potential biomarker in posterior cortical atrophy and typical Alzheimer’s disease. *Alzheimers Res Ther* **11**, 1-9.

[7] Garcia‐Martin E, Bambo MP, Marques ML, Satue M, Otin S, Larrosa JM, Polo V, Pablo LE (2016) Ganglion cell layer measurements correlate with disease severity in patients with Alzheimer's disease. *Acta Ophthalmol* **94**, e454-e459.

[8] Santangelo R, Huang S-C, Bernasconi MP, Falautano M, Comi G, Magnani G, Leocani L (2020) Neuro-retina might reflect Alzheimer’s disease stage. *J Alzheimers Dis* **77**, 1455-1468.

[9] Jáñez-Escalada L, Jáñez-García L, Salobrar-García E, Santos-Mayo A, de Hoz R, Yubero R, Gil P, Ramírez JM (2019) Spatial analysis of thickness changes in ten retinal layers of Alzheimer’s disease patients based on optical coherence tomography. *Sci Rep* **9**, 13000.

[10] Jáñez-García L, Bachtoula O, Salobrar-García E, de Hoz R, Ramirez AI, Gil P, Ramirez JM, Jáñez-Escalada L (2021) Roughness of retinal layers in Alzheimer’s disease. *Sci Rep* **11**, 11804.

[11] Ferrari L, Huang S-C, Magnani G, Ambrosi A, Comi G, Leocani L (2017) Optical coherence tomography reveals retinal neuroaxonal thinning in frontotemporal dementia as in Alzheimer’s disease. *J Alzheimers Dis* **56**, 1101-1107.

[12] Shin JY, Choi EY, Kim M, Lee HK, Byeon SH (2021) Changes in retinal microvasculature and retinal layer thickness in association with apolipoprotein E genotype in Alzheimer’s disease. *Sci Rep* **11**, 1847.

[13] Knoll B, Simonett J, Volpe NJ, Farsiu S, Ward M, Rademaker A, Weintraub S, Fawzi AA (2016) Retinal nerve fiber layer thickness in amnestic mild cognitive impairment: Case-control study and meta-analysis. *Alzheimers Dement (Amst)* **4**, 85-93.

[14] Robbins CB, Akrobetu D, Ma JP, Stinnett SS, Soundararajan S, Liu AJ, Johnson KG, Grewal DS, Fekrat S (2022) Assessment of retinal microvascular alterations in individuals with amnestic and nonamnestic mild cognitive impairment using optical coherence tomography angiography. *Retina* **42**, 1338-1346.

[15] Peng S-Y, Wu I-W, Sun C-C, Lee C-C, Liu C-F, Lin Y-Z, Yeung L (2021) Investigation of possible correlation between retinal neurovascular biomarkers and early cognitive impairment in patients with chronic kidney disease. *Transl Vis Sci Technol* **10**, 9.

[16] Montorio D, Criscuolo C, Breve MA, Lanzillo R, Salvatore E, Morra VB, Cennamo G (2022) Radial peripapillary vessel density as early biomarker in preperimetric glaucoma and amnestic mild cognitive impairment. *Graefes Arch Clin Exp Ophthalmol* **260**, 2321-2328.

[17] Almeida AL, Pires LA, Figueiredo EA, Costa-Cunha LV, Zacharias LC, Preti RC, Monteiro ML, Cunha LP (2019) Correlation between cognitive impairment and retinal neural loss assessed by swept-source optical coherence tomography in patients with mild cognitive impairment. *Alzheimers Dement (Amst)* **11**, 659-669.

[18] Un Y, Alpaslan F, Dikmen NT, Sonmez M (2022) Posterior pole analysis and ganglion cell layer measurements in Alzheimer’s disease. *Hosp Pract (1995)* **50**, 282-288.

[19] Galvin JE, Kleiman MJ, Walker M (2021) Using optical coherence tomography to screen for cognitive impairment and dementia. *J Alzheimers Dis* **84**, 723-736.

[20] Lad EM, Mukherjee D, Stinnett SS, Cousins SW, Potter GG, Burke JR, Farsiu S, Whitson HE (2018) Evaluation of inner retinal layers as biomarkers in mild cognitive impairment to moderate Alzheimer’s disease. *PLoS One* **13**, e0192646.

[21] Sánchez D, Castilla-Marti M, Marquié M, Valero S, Moreno-Grau S, Rodríguez-Gómez O, Piferrer A, Martínez G, Martínez J, Rojas ID (2020) Evaluation of macular thickness and volume tested by optical coherence tomography as biomarkers for Alzheimer’s disease in a memory clinic. *Sci Rep* **10**, 1580.

[22] den Haan J, Janssen SF, van de Kreeke JA, Scheltens P, Verbraak FD, Bouwman FH (2018) Retinal thickness correlates with parietal cortical atrophy in early-onset Alzheimer's disease and controls. *Alzheimers Dement (Amst)* **10**, 49-55.

[23] Kim BJ, Grossman M, Song D, Saludades S, Pan W, Dominguez-Perez S, Dunaief JL, Aleman TS, Ying G-S, Irwin DJ (2019) Persistent and progressive outer retina thinning in frontotemporal degeneration. *Front Neurosci* **13**, 298.

[24] Kim BJ, Irwin DJ, Song D, Daniel E, Leveque JD, Raquib AR, Pan W, Ying G-S, Aleman TS, Dunaief JL (2017) Optical coherence tomography identifies outer retina thinning in frontotemporal degeneration. *Neurology* **89**, 1604-1611.

[25] Uchida A, Pillai JA, Bermel R, Jones SE, Fernandez H, Leverenz JB, Srivastava SK, Ehlers JP (2020) Correlation between brain volume and retinal photoreceptor outer segment volume in normal aging and neurodegenerative diseases. *PLoS One* **15**, e0237078.

[26] O’Bryhim BE, Lin JB, Van Stavern GP, Apte RS (2021) OCT angiography findings in preclinical Alzheimer’s disease: 3-year follow-up. *Ophthalmology* **128**, 1489-1491.

[27] Asanad S, Fantini M, Sultan W, Nassisi M, Felix CM, Wu J, Karanjia R, Ross-Cisneros FN, Sagare AP, Zlokovic BV (2020) Retinal nerve fiber layer thickness predicts CSF amyloid/tau before cognitive decline. *PLoS One* **15**, e0232785.
