# Supplementary material for: A Systematic Review on Retinal Biomarkers to Diagnose Dementia from OCT/OCTA Images
Source: J Alzheimers Dis Rep. 2023 Nov 1;7(1):1201–35. doi: 10.3233/ADR-230042 (PMC10657718; doi:10.3233/ADR-230042)
Supplement: Supplementary Table 2 [file adr-7-adr230042-s002.docx]

**Supplementary Table 2.** Cross-Sectional Results for Vascular Parameters (Macula and Optic Disc related parameters)

|  | **SCP-FAZ** | **DCP-FAZ** | **SVC-FAZ** | **DVC-FAZ** | **IVC-FAZ** | **SVP-FAZ** | **SCP-FD** | **DCP-FD** | **SCP-VD** | **DCP-VD** | **IVC-VD** | **SVC-VD** | **DVC-VD** | **6mm SVC VD** | **6mm DVC VD** | **6mm CC- VD** | **CC-VD** | **CC-VLD** | **SCP-VLD** | **DCP-VLD** | **ICP-VLD** | **pRPC-VD** | **pRPC-VLD** | **RPC-VD W-Img** | **pSVC-VD** | **pSVC-VLD** | **OutRet-FR** |
| --- | --- | --- | --- | --- | --- | --- | --- | --- | --- | --- | --- | --- | --- | --- | --- | --- | --- | --- | --- | --- | --- | --- | --- | --- | --- | --- | --- |
| **AD** |  |  |  |  |  |  |  |  |  |  |  |  |  |  |  |  |  |  |  |  |  |  |  |  |  |  |  |
| **Supported by CSF and/or PET** |  |  |  |  |  |  |  |  |  |  |  |  |  |  |  |  |  |  |  |  |  |  |  |  |  |  |  |
| [1] ${BIOM}_{+ve}$: 9 vs. ${BIOM}_{-ve}$: 11 | $-$ | $-$ | $-$ | $-$ | $\uparrow$ | $-$ | $-$ | $-$ | $-$ | $-$ | $-$ | $-$ | $-$ | $-$ | $-$ | $-$ | $-$ | $-$ | $-$ | $-$ | $-$ | $-$ | $-$ | $-$ | $-$ | $-$ | $-$ |
| [2] AD: ${48}^{*A+}$, HCs: ${38}^{*A-}$ | $-$ | $-$ | $-$ | $-$ | $-$ | $-^{NS}$ | $-$ | $-$ | $-$ | $-$ | $-$ | $-$ | $-$ | $-$ | $-$ | $-$ | $-$ | $-$ | $-$ | $-$ | $-$ | $-$ | $-$ | $-$ | $-$ | $-$ | $-$ |
| [3] *APOE* ε4+:10 vs. *APOE* ε4-: 18 | $-^{NS}$ | $-^{NS}$ | $-$ | $-$ | $-$ | $-$ | $-$ | $-$ | $-^{NS}$ | $\downarrow$ | $-$ | $-$ | $-$ | $-$ | $-$ | $-$ | $-$ | $-$ | $-$ | $-$ | $-$ | $-$ | $-$ | $-$ | $-$ | $-$ | $-$ |
| **AD vs. HCs:** |  |  |  |  |  |  |  |  |  |  |  |  |  |  |  |  |  |  |  |  |  |  |  |  |  |  |  |
| [4] Mild AD: 37, HCs: 29 | $-$ | $-$ | $-$ | $-$ | $-$ | $-$ | $-$ | $-$ | $-$ | $-$ | $-$ | $-$ | $-$ | $-$ | $-$ | $-$ | $-$ | $-$ | $-$ | $-$ | $-$ | $\downarrow$ | $-$ | $\downarrow$ | $-$ | $-$ | $-$ |
| [5] AD: 7, HCs: 8 | $-$ | $-$ | $-$ | $-$ | $-$ | $-$ | $-$ | $-$ | $-^{NS}$ | $-^{NS}$ | $-$ | $-$ | $-$ | $-$ | $-$ | $-$ | $-^{NS}$ | $-$ | $-$ | $-$ | $-$ | $-$ | $-$ | $-$ | $-$ | $-$ | $-$ |
| [6] AD: 24 vs. HCs: 29 | $-$ | $-$ | $-$ | $-$ | $-$ | $-$ | $\downarrow$ | $-^{NS}$ | $\downarrow$ | $\downarrow$ | $-$ | $-$ | $-$ | $-$ | $-$ | $-$ | $-$ | $-$ | $-$ | $-$ | $-$ | $-$ | $-$ | $-$ | $-$ | $-$ | $-$ |
| [7] Mild AD: 17 vs. HCs: 15 | $-$ | $-$ | $-$ | $-$ | $-$ | $-$ | $-$ | $-$ | $-^{NS}$ | $-^{NS}$ | $-$ | $-$ | $-$ | $-$ | $-$ | $-$ | $-$ | $-$ | $-$ | $-$ | $-$ | $-$ | $-$ | $-$ | $-$ | $-$ | $-$ |
| [8] ATD: 26, HCs: 26 | $-$ | $-$ | $\uparrow$ | $-$ | $-$ | $-$ | $-$ | $-$ | $-$ | $-$ | $-$ | $\downarrow$ | $-$ | $-$ | $-$ | $-$ | $-$ | $-$ | $-$ | $-$ | $-$ | $-$ | $-$ | $-$ | $-$ | $-$ | $-^{NS}$ |
| **MCI** |  |  |  |  |  |  |  |  |  |  |  |  |  |  |  |  |  |  |  |  |  |  |  |  |  |  |  |
| **MCI vs. HCs:** |  |  |  |  |  |  |  |  |  |  |  |  |  |  |  |  |  |  |  |  |  |  |  |  |  |  |  |
| [9] amnestic MCI: 59 vs. non-amnestic MCI: 17 vs. HCs: 56 | $-^{NS}$ | $-$ | $-$ | $-$ | $-$ | $-$ | $-$ | $-$ | $-^{NS}$ | $-$ | $-$ | $-$ | $-$ | $-$ | $-$ | $-$ | $-$ | $-$ | $-^{NS}$ | $-$ | $-$ | $-$ | $-$ | $-$ | $-$ | $-$ | $-$ |
| [9] non-amnestic MCI: 17 vs. HCs: 56 | $-^{NS}$ | $-$ | $-$ | $-$ | $-$ | $-$ | $-$ | $-$ | $-^{NS}$ | $-$ | $-$ | $-$ | $-$ | $-$ | $-$ | $-$ | $-$ | $-$ | $-^{NS}$ | $-$ | $-$ | $-$ | $-$ | $-$ | $-$ | $-$ | $-$ |
| [9] amnestic MCI: 59 vs. HCs: 56 | $-^{NS}$ | $-$ | $-$ | $-$ | $-$ | $-$ | $-$ | $-$ | $-^{NS}$ | $-$ | $-$ | $-$ | $-$ | $-$ | $-$ | $-$ | $-$ | $-$ | $-^{NS}$ | $-$ | $-$ | $-$ | $-$ | $-$ | $-$ | $-$ | $-$ |
| [9] amnestic MCI: 59 vs. non-amnestic MCI: 17 | $-^{NS}$ | $-$ | $-$ | $-$ | $-$ | $-$ | $-$ | $-$ | $-^{NS}$ | $-$ | $-$ | $-$ | $-$ | $-$ | $-$ | $-$ | $-$ | $-$ | $-^{NS}$ | $-$ | $-$ | $-$ | $-$ | $-$ | $-$ | $-$ | $-$ |
| [6] MCI: 37 vs. HCs: 29 | $-$ | $-$ | $-$ | $-$ | $-$ | $-$ | $\downarrow$ | $-^{NS}$ | $-$ | $-$ | $-$ | $-$ | $-$ | $-$ | $-$ | $-$ | $-$ | $-$ | $\downarrow$ | $-^{NS}$ | $-$ | $-$ | $-$ | $-$ | $-$ | $-$ | $-$ |
| [10] MCI due to AD: 24 vs. HCs: 13 | $-^{NS}$ | $-$ | $-$ | $-$ | $-$ | $-$ | $-$ | $-$ | $-^{NS}$ | $\downarrow$ | $\downarrow$ | $-$ | $-$ | $-$ | $-$ | $-$ | $-^{NS}$ | $-^{NS}$ | $\downarrow$ | $\downarrow$ | $\downarrow$ | $-$ | $-$ | $-$ | $-$ | $-$ | $-$ |
| [3] MCI: 24 vs. HCs: 31 | $-^{NS}$ | $\uparrow$ | $-$ | $-$ | $-$ | $-$ | $-$ | $-$ | $\downarrow$ | $-^{NS}$ | $-$ | $-$ | $-$ | $-$ | $-$ | $-$ | $-$ | $-$ | $-$ | $-$ | $-$ | $-$ | $-$ | $-$ | $-$ | $-$ | $-$ |
| [11] CI: 268, HCs: 1287 | $-$ | $-^{NS}$ | $-$ | $-$ | $-$ | $-$ | $-$ | $-$ | $-$ | $-$ | $-$ | $-$ | $-$ | $-$ | $-$ | $-$ | $-$ | $-$ | $-$ | $-$ | $-$ | $-$ | $-$ | $-$ | $-$ | $-$ | $-$ |
| [12] CKD_Low: 13 vs. CKD_High: 99 | $-$ | $-$ | $-$ | $-$ | $-^{NS}$ | $-$ | $-$ | $-$ | $-$ | $-$ | $-$ | $-$ | $-$ | $-$ | $-$ | $-$ | $-$ | $-$ | $-$ | $-$ | $-$ | $-$ | $-$ | $-$ | $-$ | $-$ | $-$ |
| [13] aMCI: 54 vs. HCs: 54 | $-$ | $-$ | $-$ | $-$ | $-$ | $-$ | $-$ | $-$ | $-$ | $-$ | $-$ | $-$ | $-$ | $-$ | $-$ | $-$ | $-$ | $-$ | $-$ | $-$ | $-$ | $-$ | $-$ | $-^{NS}$ | $-$ | $-$ | $-$ |
| **Multi-Class comparison** |  |  |  |  |  |  |  |  |  |  |  |  |  |  |  |  |  |  |  |  |  |  |  |  |  |  |  |
| [14] MCI: 21 vs. AD: 18 vs. HCs: 21 | $-$ | $-$ | $-$ | $-$ | $\uparrow$ | $-$ | $-$ | $-$ | $-$ | $-$ | $-$ | $-$ | $-$ | $-$ | $-$ | $-$ | $-$ | $-$ | $-$ | $-$ | $-$ | $-$ | $-$ | $-$ | $-$ | $-$ | $-$ |
| [15] aMCI/eAD: 16 vs. HCs: 16 | $-$ | $-$ | $-$ | $-$ | $-^{NS}$ | $-$ | $-$ | $-$ | $-$ | $-$ | $-$ | $-$ | $-$ | $-$ | $-$ | $-$ | $-$ | $-$ | $-$ | $-$ | $-$ | $-$ | $-^{NS}$ | $-$ | $-^{NS}$ | $-^{NS}$ | $-$ |
| [16] AD: 12, MCI: 12, HCs: 32 | $-$ | $-$ | $-$ | $-$ | $-$ | $-$ | $-$ | $-$ | $-$ | $-$ | $-$ | $-^{NS}$ | $-^{NS}$ | $-^{NS}$ | $-^{NS}$ | $-^{NS}$ | $-^{NS}$ | $-$ | $-$ | $-$ | $-$ | $-$ | $-$ | $-$ | $-$ | $-$ | $-$ |

Noteworthy, all tables illustrate significant changes by $\uparrow$ or $\downarrow$ arrows, insignificant changes by $-^{NS}$, and $-$ for any missing parameters.

See Table 2 for definitions of abbreviations.

**REFERENCES**

[1] O’Bryhim BE, Lin JB, Van Stavern GP, Apte RS (2021) OCT angiography findings in preclinical Alzheimer’s disease: 3-year follow-up. *Ophthalmology* **128**, 1489-1491.

[2] den Haan J, van de Kreeke JA, van Berckel BN, Barkhof F, Teunissen CE, Scheltens P, Verbraak FD, Bouwman FH (2019) Is retinal vasculature a biomarker in amyloid proven Alzheimer's disease? *Alzheimers Dement (Amst)* **11**, 383-391.

[3] Shin JY, Choi EY, Kim M, Lee HK, Byeon SH (2021) Changes in retinal microvasculature and retinal layer thickness in association with apolipoprotein E genotype in Alzheimer’s disease. *Sci Rep* **11**, 1847.

[4] Yan Y, Wu X, Wang X, Geng Z, Wang L, Xiao G, Wu Y, Zhou S, Liao R, Wei L (2021) The retinal vessel density can reflect cognitive function in patients with Alzheimer’s disease: evidence from optical coherence tomography angiography. *J Alzheimers Dis* **79**, 1307-1316.

[5] Sadda SR, Borrelli E, Fan W, Ebraheem A, Marion KM, Harrington M, Kwon S (2019) A pilot study of fluorescence lifetime imaging ophthalmoscopy in preclinical Alzheimer’s disease. *Eye* **33**, 1271-1279.

[6] Chua J, Hu Q, Ke M, Tan B, Hong J, Yao X, Hilal S, Venketasubramanian N, Garhöfer G, Cheung CY (2020) Retinal microvasculature dysfunction is associated with Alzheimer’s disease and mild cognitive impairment. *Alzheimers Res Ther* **12**, 161.

[7] Salobrar-Garcia E, Méndez-Hernández C, Hoz Rd, Ramírez AI, López-Cuenca I, Fernández-Albarral JA, Rojas P, Wang S, García-Feijoo J, Gil P (2020) Ocular vascular changes in mild alzheimer’s disease patients: Foveal avascular zone, choroidal thickness, and onh hemoglobin analysis. *J Person Med* **10**, 231.

[8] Bulut M, Kurtuluş F, Gözkaya O, Erol MK, Cengiz A, Akıdan M, Yaman A (2018) Evaluation of optical coherence tomography angiographic findings in Alzheimer’s type dementia. *Br J Ophthalmol* **102**, 233-237.

[9] Robbins CB, Akrobetu D, Ma JP, Stinnett SS, Soundararajan S, Liu AJ, Johnson KG, Grewal DS, Fekrat S (2022) Assessment of retinal microvascular alterations in individuals with amnestic and nonamnestic mild cognitive impairment using optical coherence tomography angiography. *Retina* **42**, 1338-1346.

[10] Biscetti L, Lupidi M, Luchetti E, Eusebi P, Gujar R, Vergaro A, Cagini C, Parnetti L (2021) Novel noninvasive biomarkers of prodromal Alzheimer disease: The role of optical coherence tomography and optical coherence tomography–angiography. *Eur J Neurol* **28**, 2185-2191.

[11] Yang K, Cui L, Chen X, Yang C, Zheng J, Zhu X, Xiao Y, Su B, Li C, Shi K (2022) Decreased vessel density in retinal capillary plexus and thinner ganglion cell complex associated with cognitive impairment. *Front Aging Neurosci* **14**, 872466.

[12] Peng S-Y, Wu I-W, Sun C-C, Lee C-C, Liu C-F, Lin Y-Z, Yeung L (2021) Investigation of possible correlation between retinal neurovascular biomarkers and early cognitive impairment in patients with chronic kidney disease. *Transl Vis Sci Technol* **10**, 9.

[13] Montorio D, Criscuolo C, Breve MA, Lanzillo R, Salvatore E, Morra VB, Cennamo G (2022) Radial peripapillary vessel density as early biomarker in preperimetric glaucoma and amnestic mild cognitive impairment. *Graefes Arch Clin Exp Ophthalmol* **260**, 2321-2328.

[14] Wu J, Zhang X, Azhati G, Li T, Xu G, Liu F (2020) Retinal microvascular attenuation in mental cognitive impairment and Alzheimer's disease by optical coherence tomography angiography. *Acta Ophthalmol* **98**, e781-e787.

[15] Zhang YS, Zhou N, Knoll BM, Samra S, Ward MR, Weintraub S, Fawzi AA (2019) Parafoveal vessel loss and correlation between peripapillary vessel density and cognitive performance in amnestic mild cognitive impairment and early Alzheimer’s disease on optical coherence tomography angiography. *PLoS One* **14**, e0214685.

[16] Querques G, Borrelli E, Sacconi R, De Vitis L, Leocani L, Santangelo R, Magnani G, Comi G, Bandello F (2019) Functional and morphological changes of the retinal vessels in Alzheimer’s disease and mild cognitive impairment. *Sci Rep* **9**, 63.
