# Supplementary material for: A Systematic Review on Retinal Biomarkers to Diagnose Dementia from OCT/OCTA Images
Source: J Alzheimers Dis Rep. 2023 Nov 1;7(1):1201–35. doi: 10.3233/ADR-230042 (PMC10657718; doi:10.3233/ADR-230042)
Supplement: Supplementary Table 3 [file adr-7-adr230042-s003.docx]

**Supplementary Table 3.** Longitudinal Macula-related parameters

|  | FRT | ILM | mRNFL | GCL | IPL | mRNFL + GCL | GC-IPL | GCC | INL | OPL | ONL | ELM/ OLM | PhotoR | IS-OS/EZ | OSL | IZ | RPE | OPR | Macula volume | GCL Volume | IPL Volume | INL Volume | Outer Retina | Other Special Parameters |
| --- | --- | --- | --- | --- | --- | --- | --- | --- | --- | --- | --- | --- | --- | --- | --- | --- | --- | --- | --- | --- | --- | --- | --- | --- |
| [1] AD <3 years: 99 vs. AD ≥3 years: 51 | $-$ | $-^{\boldsymbol{NS}}$ | $\boldsymbol{\downarrow}$ | $\boldsymbol{\downarrow}$ | $\boldsymbol{\downarrow}$ | $-$ | $-$ | $-$ | $\mathbf{-}^{\boldsymbol{NS}}$ | $\mathbf{-}^{\boldsymbol{NS}}$ | $\mathbf{-}^{\boldsymbol{NS}}$ | $\mathbf{-}^{\boldsymbol{NS}}$ | $\mathbf{-}^{\boldsymbol{NS}}$ | $-$ | $-$ | $-$ | $\mathbf{-}^{\boldsymbol{NS}}$ | $-$ | $-$ | $-$ | $-$ | $-$ | $-$ |  |
| [1] HCs: 75 vs. AD <3 years: 99 vs. AD ≥3 years: 51 | $-$ | $-^{\boldsymbol{NS}}$ | $\boldsymbol{\downarrow}$ | $\boldsymbol{\downarrow}$ | $\boldsymbol{\downarrow}$ | $-$ | $-$ | $-$ | $\mathbf{-}^{\boldsymbol{NS}}$ | $\mathbf{-}^{\boldsymbol{NS}}$ | $\mathbf{-}^{\boldsymbol{NS}}$ | $\mathbf{-}^{\boldsymbol{NS}}$ | $\mathbf{-}^{\boldsymbol{NS}}$ | $-$ | $-$ | $-$ | $\mathbf{-}^{\boldsymbol{NS}}$ | $-$ | $-$ | $-$ | $-$ | $-$ | $-$ |  |
| [2] aMCI: 19  2 years follow-up vs. baseline. | $-$ | $-$ | $-$ | $-$ | $-$ | $-$ | $-$ | $\downarrow$ | $-$ | $-$ | $-$ | $-$ | $-$ | $-$ | $-$ | $-$ | $-$ | $-$ | $-$ | $-$ | $-$ | $-$ | $-$ |  |
| [3] FTD: 16 vs. HCs: 30 $*^{\aleph2}$ (Annual changes) | $-^{NS}$ | $-$ | $-^{NS}$ | $-^{NS}$ | $-^{NS}$ | $-$ | $-$ | $-$ | $-^{NS}$ | $-^{NS}$ | $-^{NS}$ | $-$ | $-^{NS}$ | $-^{NS}$ | $-$ | $-^{NS}$ | $-^{NS}$ | $-$ | $-$ | $-$ | $-$ | $-$ | $-^{NS}$ |  |
| [3] FTD: 16 vs. HCs: 30 $*^{AdjX}$ (Annual changes) | $-^{NS}$ | $-$ | $-^{NS}$ | $-^{NS}$ | $-^{NS}$ | $-$ | $-$ | $-$ | $\downarrow$ | $-^{NS}$ | $-^{NS}$ | $-$ | $-^{NS}$ | $-^{NS}$ | $-$ | $-^{NS}$ | $-^{NS}$ | $-$ | $-$ | $-$ | $-$ | $-$ | $-^{NS}$ |  |
| [4] $MCI_{AD}:$ 9 vs. $MCI_{MCI}$: 12 | $-^{NS}$ | $-$ | $-$ | $-$ | $-$ | $-$ | $\downarrow$ | $-$ | $-$ | $-$ | $-$ | $-$ | $-$ | $-$ | $-$ | $-$ | $-$ | $-$ | $-^{NS}$ | $-$ | $-$ | $-$ | $-$ | MT C, GC-IPL S, GC-IPL ST, GC-IPL SN: $-^{NS}.$GC-IPL min, GC-IPL I, GC-IPL IN, GC-IPL IT: $\downarrow$ |

$*^{AdjX}$, linear models are adjusted for age, sex, and race, ℵ2 The study by [3] had an average follow-up of 14.5- and 15.6-months for HCs and FTD, correspondingly.

Noteworthy, all tables illustrate significant changes by $\uparrow$ or $\downarrow$ arrows, insignificant changes by $-^{NS}$, and $-$ for any missing parameters.

**REFERENCES**

[1] Garcia‐Martin E, Bambo MP, Marques ML, Satue M, Otin S, Larrosa JM, Polo V, Pablo LE (2016) Ganglion cell layer measurements correlate with disease severity in patients with Alzheimer's disease. *Acta Ophthalmol* **94**, e454-e459.

[2] Criscuolo C, Cennamo G, Montorio D, Carotenuto A, Migliaccio M, Moccia M, Salvatore E, Lanzillo R, Costagliola C, Brescia Morra V (2022) A two-year longitudinal study of retinal vascular impairment in patients with amnestic mild cognitive impairment. *Front Aging Neurosci* **14**, 993621.

[3] Kim BJ, Grossman M, Song D, Saludades S, Pan W, Dominguez-Perez S, Dunaief JL, Aleman TS, Ying G-S, Irwin DJ (2019) Persistent and progressive outer retina thinning in frontotemporal degeneration. *Front Neurosci* **13**, 298.

[4] Choi SH, Park SJ, Kim NR (2016) Macular ganglion cell-inner plexiform layer thickness is associated with clinical progression in mild cognitive impairment and Alzheimers disease. *PLoS One* **11**, e0162202.
